# Itgb1‐Mediated Stabilization of Vimentin Alleviates Excessive Mechanical Stress‐Induced Nucleus Pulposus Cell Pyroptosis and Intervertebral Disc Degeneration via PINK1‐Parkin‐Dependent Mitophagy

**DOI:** 10.1111/cpr.70256

**Published:** 2026-07-14

**Authors:** Xuening Liu, Fengguang Yang, Yanni Duan, Hefang Xiao, Zhenyu Cao, Zhaoheng Wang, Haijun Zhang, Xuewen Kang

**Affiliations:** ^1^ Department of Orthopedics Lanzhou University Second Hospital Lanzhou People's Republic of China; ^2^ The Second Clinical Medical College Lanzhou University Lanzhou People's Republic of China; ^3^ Key Laboratory of Orthopedic Disease of Gansu Province Lanzhou University Second Hospital Lanzhou People's Republic of China

**Keywords:** intervertebral disc degeneration, Itgb1, mitophagy, pyroptosis, vimentin

## Abstract

Excessive mechanical stress is a main cause of intervertebral disc degeneration (IDD). However, the specific mechanism remains unclear. We established in vivo and in vitro models to investigate the role of cytoskeletal proteins in excessive mechanical stress‐induced NP cell pyroptosis and IDD. The expression level of Vimentin was decreased in degenerated NP cells induced by excessive mechanical stress. Knockdown of Vimentin promoted NP cell pyroptosis and IDD in rats, whereas Vimentin overexpression significantly alleviated excessive mechanical stress‐induced NP cell pyroptosis and degeneration. Further mechanistic studies revealed that Vimentin ameliorated mitochondrial dysfunction triggered by excessive mechanical stress through PINK1‐Parkin‐dependent mitophagy, thereby attenuating NP cell pyroptosis and degeneration. Co‐immunoprecipitation‐mass spectrometry analysis suggested an interaction between Itgb1 and Vimentin, which was validated by Co‐immunoprecipitation assays. Itgb1 enhanced Vimentin protein stability via the ubiquitin‐proteasome pathway and inhibited mechanical stress‐mediated Vimentin degradation. Subsequent experiments confirmed that Itgb1 reduced Vimentin ubiquitination and degradation by blocking the binding of MNAT1 to Vimentin. Itgb1 ameliorated excessive mechanical stress‐induced NP cell pyroptosis and degeneration via Vimentin. Restoring Vimentin function through gene overexpression effectively inhibited NP cell pyroptosis and delayed the progression of IDD in rats. In summary, this study reveals a mechanotransduction pathway from mechanical stress sensing to cellular functional regulation in IDD, providing novel insights into the pathological mechanisms underlying IDD. Moreover, this study demonstrates that Vimentin exerts a significant protective effect against excessive mechanical stress‐induced NP cell pyroptosis and IDD, offering a potential therapeutic target for the clinical management of IDD.

## Graphical abstract

Excessive mechanical stress induces nucleus pulposus cell pyroptosis and intervertebral disc degeneration. Itgb1 inhibits MNAT1‐mediated ubiquitination and degradation of Vimentin. Vimentin alleviates excessive mechanical stress‐induced nucleus pulposus cell pyroptosis and intervertebral disc degeneration through PINK1‐Parkin‐dependent mitophagy.

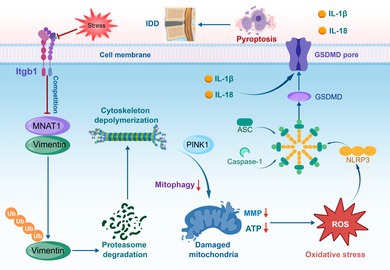

## 1 Introduction

Low back pain is a common musculoskeletal disorder with a prevalence rate of ~80% [1]. Intervertebral disc degeneration (IDD) is one of the primary causes of low back pain, affecting ~90% of the population aged 50–55 years [2]. Despite the high prevalence of IDD, the precise pathogenesis remains poorly understood, limiting the identification of effective therapeutic targets. A better understanding of the underlying pathological mechanisms of IDD will facilitate the identification of biomarker‐driven therapeutic targets and the development of new strategies for preventing and treating this condition.

The intervertebral disc (IVD) is a fibrocartilaginous structure responsible for resisting compression [3]. The IVD is subjected to different loads under various postures, ranging from 0.1 to 0.2 MPa in the supine position, 0.4–0.5 MPa in the standing position, and 0.8–1.0 MPa in the forward‐leaning sitting position [4]. The nucleus pulposus (NP) is a proteoglycan‐rich, gelatinous, and highly hydrated tissue that serves as an essential “cushion” for the spine [5]. During movement in various directions or under load, the NP deforms accordingly, enabling the distribution of mechanical stress along the spine [6]. Accumulating evidence suggests that excessive mechanical stress exacerbates IDD by disrupting the balance between anabolism and catabolism within the NP [7]. However, the potential molecular mechanism is still unclear.

Pyroptosis, a highly inflammatory form of programmed cell death, is a key cellular event contributing to the onset and progression of IDD [8]. NP cell pyroptosis is initiated by the activation of the inflammasome (NLRP3) and gasdermin family proteins (GSDMD), leading to pore formation in the NP cell membrane, cell swelling, lytic death, and the release of pro‐inflammatory cytokines such as IL‐1β and IL‐18 [9]. These cytokines exacerbate extracellular matrix (ECM) catabolism by upregulating matrix‐degrading enzymes (including MMPs and ADAMTSs), while simultaneously inhibiting the synthesis of collagen and aggrecan [10]. However, the specific molecular mechanism underlying NP cell pyroptosis in IDD induced by excessive mechanical stress remains not fully understood and requires further elucidation.

Mitochondria are highly dynamic organelles that enable cells to adapt and respond to a changing microenvironment through continuous fusion and fission [11]. Mitochondrial dysfunction in NP cells has been confirmed to be associated with IDD [12, 13]. Dysfunctional mitochondria induce the production of reactive oxygen species (ROS). ROS can activate the NLRP3 inflammasome to facilitate nucleus pulposus cell pyroptosis and accelerate extracellular matrix degradation [14, 15]. Mitophagy serves as a vital mitochondrial quality control mechanism, which specifically recognizes damaged mitochondria and eliminates them via lysosomes. Accumulating evidence has demonstrated that impaired autophagy is closely associated with the progression of IDD [16, 17]. Nevertheless, the mechanism by which mitophagy participates in IDD induced by excessive mechanical stress remains unclear.

Vimentin is one of the most abundant intermediate filament proteins, playing a vital role in stress protection and mechanical support [18]. The cytoskeleton can regulate the morphology and function of mitochondria [19, 20]. Vimentin‐deficient cells exhibit mitochondrial fragmentation and disorganization [21]. The expression level of Vimentin is reduced in degenerated NP tissues [22, 23]. Moreover, Vimentin is often subjected to post‐translational modifications such as phosphorylation, ubiquitination, and acetylation, which regulate various biological behaviours [24]. CircARPC1B inhibits Vimentin degradation through the ubiquitin‐proteasome pathway, alleviating high cholesterol‐induced osteoarthritis [25]. These results suggest that Vimentin exerts potential protective effects during IDD. However, no in vivo or in vitro experiments have yet explored whether Vimentin can protect against excessive mechanical stress‐induced IDD and elucidate the underlying molecular mechanisms.

In this study, we found that the level of Vimentin in NP cells was decreased under excessive mechanical stress. Vimentin alleviated excessive mechanical stress‐induced NP cell pyroptosis and IDD through PINK1‐Parkin‐dependent mitophagy. Furthermore, we found that Itgb1 inhibited excessive mechanical stress‐induced Vimentin degradation via the ubiquitin‐proteasome pathway. Itgb1 alleviated excessive mechanical stress‐induced mitophagy impairment, pyroptosis, and ECM degradation in NP cells through Vimentin. This study reveals a new mechanism of mechanical stress‐induced IDD. This may provide a new potential therapeutic target for IDD.

## 2 Methods and Materials

### 2.1 Human NP Tissue Samples

Human NP samples were obtained from patients undergoing spinal surgery at the Lanzhou University Second Hospital (Lanzhou, China). The degree of degeneration was evaluated by two independent physicians who were blinded to group assignments according to the MRI‐based Pfirrmann grading system [26]. Any inconsistent scores were adjudicated by a third physician. NP specimens graded as Pfirrmann II/III (*n* = 6; age range: 28–40 years) were classified as the mild IDD group, while specimens graded as Pfirrmann IV/V (*n* = 6; age range: 46–62 years) were classified as the severe IDD group. This study was approved by the Ethics Committee of the Second Hospital of Lanzhou University (Approval No. 2025A‐559), and written informed consent was obtained from each donor.

### 2.2 Animal Experiments

Male Sprague–Dawley rats (8 weeks old) were purchased from the Experimental Animal Center of Lanzhou University (Lanzhou, China). All animal experiments were approved by the Animal Ethics Committee of the Second Hospital of Lanzhou University (Approval No. D2025‐427). As described in previous studies, tail compression suture (TCS) was adopted to establish the rat IDD model [27]. In the sham group, only a 5‐mm‐wide skin strip was removed without suture. All rats underwent imaging and histological examinations 10 weeks after surgery. To evaluate the effect of Vimentin knockdown or overexpression on IDD, lentivirus (10 μL) was injected directly into the NP of the IVDs using a 33‐gauge needle. Rats were injected with an equivalent volume of 0.9% sodium chloride solution as a control. All animals were housed under specific pathogen‐free conditions with ad libitum access to food and water, and were allowed unrestricted activity.

### 2.3 Primary Rat NP Cell Isolation, Culture, and In Vitro Compression System

Six male Sprague–Dawley rats were euthanized via intraperitoneal injection of an overdose of pentobarbital. Tails were aseptically removed. Gelatinous NP tissue was carefully dissected. The harvested NP tissue was digested in 2 mg/mL collagenase II (Biosharp, BS164) for 30 min. Isolated cells were maintained in DMEM/F12 medium (Gibco, 12,634) supplemented with 10% fetal bovine serum (FBS; Biosharp, BL1047B) and 1% penicillin–streptomycin (Biosharp, BL505A) and cultured at 37°C with 5% CO_2_. To simulate the excessive mechanical stress experienced by human NP cells, NP cells were subjected to compression using a cell compression device (CellScale, MechanoCulture TR). NP cells were compressed at 1 MPa with a frequency of 1 Hz for 8 h.

In separate pharmacological experiments, NP cells were treated with 3‐MA (5 mM; Selleck, S2767), cycloheximide (CHX, 10 μM; Selleck, S7418), or MG132 (10 μM; Selleck, S2619).

### 2.4 Lentiviral Transduction and In Vitro siRNA

Lentiviral vectors for Vimentin or Itgb1 overexpression and knockdown were produced by Genechem (China). Transduction was performed according to the manufacturer's instructions. After 24 h of transfection, cells were treated with puromycin (10 μM; Selleck, E4996) for selection. Cells were then subjected to pharmacological treatments or in vitro compression 48 h post‐transduction. siRNA duplexes targeting rat PINK1 and MNAT1 were designed and synthesized by GenePharma (China). The sequence of siRNA is as follows: PINK1(5′‐GCTGCAATGCCGCTGTGTA‐3′) and MNAT1(5′‐GGAAGCAUUGGAGGUAGAATT‐3′). Cells were transfected using the siRNA‐mate plus transduction kit (GenePharma, G04026) according to the manufacturer's instructions. Cells were subjected to pharmacological treatments or in vitro compression after 48 h.

### 2.5 X‐Ray and MRI Analysis

X‐ray imaging was performed using the following exposure parameters: exposure time, 0.06 s; source‐to‐detector distance, 100 cm; tube current, 160 mA; tube voltage, 50 kV. MRI scans were acquired using a 3.0 Tesla system. Volumetric sagittal T2‐weighted images were obtained with the following parameters: repetition time (TR) = 6000 ms; echo time (TE) = 90 ms; field of view (FOV) = 200 × 200 mm; slice thickness = 2 mm. The water content of NP was quantified by calculating the average grayscale value using ImageJ software.

### 2.6 Histology and Immunostaining Assays

Human NP tissues were immediately fixed in 4% paraformaldehyde for 48 h. The rat IVDs were fixed in 4% paraformaldehyde for 48 h and then decalcified in 10% ethylenediaminetetraacetic acid for 8 weeks. Tissues were subsequently dehydrated, embedded in paraffin, and sectioned at a thickness of 5 μm. Sections were stained with Haematoxylin and Eosin (H&E), Safranin O and Fast Green (SO&FG), and Alcian Blue to evaluate histological features. The degree of IDD was assessed using an IVD histological grading system [28].

For immunohistochemistry (IHC) staining, tissue sections were deparaffinized and rehydrated. Antigen retrieval was performed using citrate buffer (0.1 mol·L^−1^, pH 6.0). Sections were then incubated with primary antibodies overnight at 4°C after endogenous peroxidase activity was blocked. Sections were incubated with biotinylated secondary antibodies. Immunoreactivity was visualized using a 3,3′‐diaminobenzidine peroxidase substrate kit. Sections were counterstained with haematoxylin, dehydrated, cleared in xylene, and mounted with neutral balsam. Detailed antibody information is listed in Supplementary Table 1.

For immunofluorescence (IF) staining, tissue sections were prepared identically to the IHC protocol through the antigen retrieval step. Sections were blocked with 10% goat serum (Solarbio, SL038) and incubated overnight at 4°C with primary antibodies. The sections were incubated with Alexa Fluor 488‐ or 594‐conjugated secondary antibodies, and then the cell nuclei were stained with DAPI solution (Beyotime, P0131‐5 mL). Detailed antibody information is listed in Supplementary Table 1.

For cell slide staining, NP cells were fixed with 4% paraformaldehyde for 20 min. Cells were permeabilized and blocked according to standard protocols. Cells were incubated overnight at 4°C with the primary antibody. Cells were then incubated for 1 h with appropriate secondary antibodies. The cell nuclei were stained with DAPI solution. Detailed antibody information is listed in Supplementary Table 1.

The sections or slides were observed using a fluorescence microscope (Olympus, BX53). Images were analysed using ImageJ software.

### 2.7 Protein Extraction, Western Blot Analysis, and Co‐Immunoprecipitation (Co‐IP)

Following experimental interventions, NP cells were harvested and lysed in RIPA lysis buffer (Beyotime, P0013) supplemented with protease and phosphatase inhibitors. Equal amounts of protein lysates were separated by SDS‐PAGE and electrophoretically transferred onto PVDF membranes. Membranes were blocked and incubated with the primary antibody or secondary antibodies (1:5000; ZSGB, ZS2301/ZS2305). Protein bands were visualized using a ChemiDoc MP Imaging System (Bio‐Rad, 12,003,154). Band intensities were quantified using ImageJ software. Detailed antibody information is listed in Supplementary Table 1.

NP cell lysates were incubated with the primary antibody with Protein A/G magnetic beads at 4°C overnight on a rotator. Following incubation, the beads were washed five times with ice‐cold NP‐40 lysis buffer. The immunoprecipitated protein complexes were eluted and analysed by Western blot.

### 2.8 Transmission Electron Microscopy

NP cells were fixed in 2.5% glutaraldehyde (Sigma Aldrich, USA) for 1 h and then treated with 2% osmium tetroxide for 2 h, followed by dehydration in ethanol. Samples were then embedded in epoxy resin. Ultrathin sections (70–90 nm thickness) were cut using an ultramicrotome (EM UC7, Leica). Sections were observed using a Tecnai G2 TWIN transmission electron microscope (FEI, USA).

### 2.9 Mitochondrial Membrane Potential Analysis

NP cells were incubated with 500 μL of JC‐1 working solution (Beyotime, C2006) for 20 min at 37°C in the dark. Fluorescence images were captured using a fluorescence microscope. ImageJ software was used to quantify fluorescence intensities. MMP was expressed as the ratio of red fluorescence intensity to green fluorescence intensity.

### 2.10 ROS and Mitochondrial Superoxide Detection

NP cells were incubated with DCFH‐DA working solution (Beyotime, S0033) at 37°C for 20 min in the dark. Fluorescent images were captured using a fluorescence microscope (Olympus, BX53). Fluorescence intensity was quantified using ImageJ software. NP cells were incubated with MitoSOX Red working solution (Beyotime, S0061S) at 37°C for 30 min in the dark. Fluorescence intensity was measured using flow cytometry (Beckman, USA). Data were analysed using FlowJo software.

### 2.11 Lactate Dehydrogenase Assay

The level of lactate dehydrogenase (LDH) reflects the integrity of the cell membrane. Culture supernatants collected from NP cells were incubated with the LDH working solution (Beyotime, C0018S) for 30 min. Absorbance was measured at 450 nm using a microplate reader (BioTek, USA).

### 2.12 ATP Content Determination

ATP content was measured using the ATP assay kit (Beyotime, S0026) according to the manufacturer's instructions. The ATP levels were measured using the firefly luciferase method. The ATP content in the sample was calculated using a standard curve.

### 2.13 Statistical Analysis

Data are presented as the mean ± SD of at least three independent experiments. For comparisons between two groups, a *t*‐test was applied. Comparisons among multiple groups were performed using one‐way or two‐way ANOVA, followed by Tukey's post hoc test. All statistical analyses were conducted using GraphPad Prism 8 software. *p* < 0.05 was considered statistically significant.

## 3 Results

### 3.1 Excessive Mechanical Stress Induced Pyroptosis and Led to Decreased Expression of Vimentin in NP Tissues

**FIGURE 1:**
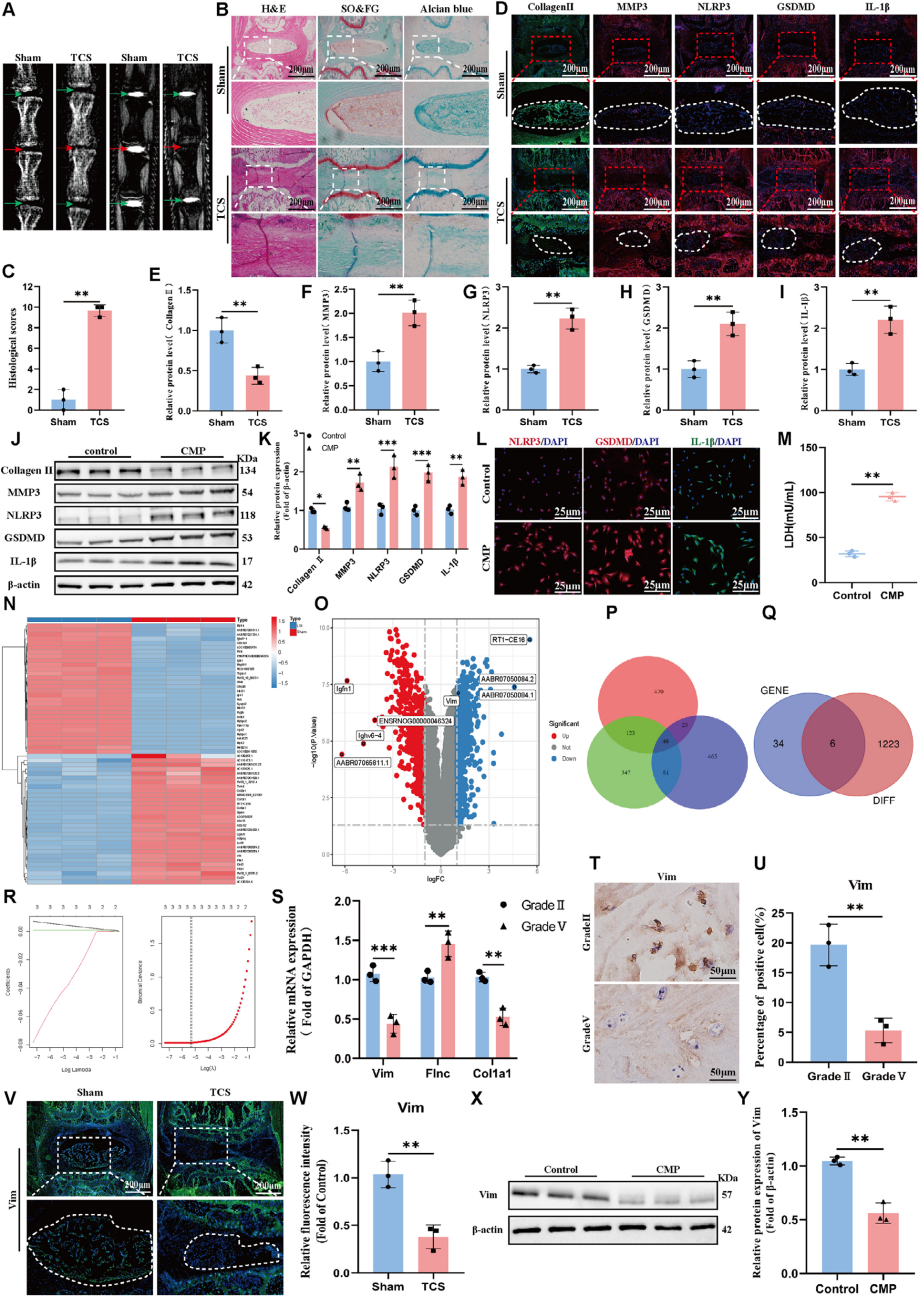
Excessive mechanical stress induced pyroptosis and led to decreased expression of Vimentin in NP tissues. (A) Representative images of X‐ray and MRI at the T2‐weighted sequence of coccygeal IVDs in rats, the green arrows indicate the control disc, and the red arrows indicate the surgical disc. (B, C) H&E, SO&FG, and Alcian blue staining in coccygeal IVDs of rats (*n* = 3). Scale bar: 200 μm. (D–I) Immunofluorescence staining of NP tissues in coccygeal IVDs of rats (*n* = 3). Scale bar: 200 μm. (J, K) The protein expressions levels in NP cell with or without compression treatment were detected by western blotting (*n* = 3). (L) Immunofluorescence staining of NLRP3, GSDMD, and IL‐1β in NP cell with or without compression. Scale bar: 25 μm. (M) The level of LDH in NP cell with or without compression (*n* = 3). (N) Heatmap of differentially expressed genes in GSE266883 data. (O) Volcano plot of differentially expressed genes in GSE266883 data. (P, Q) The Venn diagram of the mechanotransduction‐related genes (40 common genes) in GeneCards and differentially expressed genes from the GSE266883 dataset yielded 6 co‐expressed key differentially expressed genes (Vimentin, Flnc, Fn1, Gja1, Col1a1, and Mmp14). (R) The accuracy of bioinformatics analysis was improved through the LASSO regression model, resulting in three differentially expressed genes (Vimentin, Flnc, and Col1a1). (S) The mRNA expression levels of Vimentin, Flnc, and Col1a1 in human NP tissues with different degrees of degeneration were determined by qRT‐PCR (*n* = 3). (T, U) Immunohistochemical staining of Vimentin in human NP tissues with different degrees of degeneration (*n* = 3). Scale bar: 50 μm. (V, W) Immunofluorescence staining of Vimentin in NP tissues of rat coccygeal IVDs (*n* = 3). Scale bar: 200 μm. (X, Y) The protein expression of Vimentin in NP cells with or without compression was determined by western blotting (*n* = 3). Data are represented as mean ± SD. *p* value was calculated with *t*‐test. *p < 0.05, **p < 0.01, ***p < 0.001.

We detected the levels of pyroptosis in human degenerated NP tissue. The expression levels of NLRP3, GSDMD, and IL‐1β were increased in grade V compared with grade II (Figure S1A–E). To investigate the effect of excessive mechanical stress on NP cell pyroptosis, we established a rat IDD model by tail compression suture (TCS). The disc height index (DHI) and NP water content were decreased in the TCS rats (Figure 1A; Figure S1F,G), and were accompanied by increased histological scores (Figure 1B,C). Immunofluorescence staining demonstrated that the number of cells positive for NLRP3, GSDMD, and IL‐1β was increased in the TCS group (Figure 1D–I). To simulate the excessive mechanical stress experienced by human NP cells, NP cells were subjected to compression using a cell compression device. The vitality of NP cells was decreased under compression (Figure S1H). The anabolic decreased while the catabolic increased under compression (Figure S1I,J). The cytoskeletal structure of NP cells was disrupted under compression (Figure S1K). Western blot and immunofluorescence staining showed that the levels of NLRP3, GSDMD, and IL‐1β were elevated in the compression group (Figure 1J–L). LDH release increased in NP cells under compression (Figure 1M). These results collectively indicate that excessive mechanical stress induced NP cell pyroptosis.

To further investigate the molecular mechanisms underlying NP cell pyroptosis induced by excessive mechanical stress, we analysed transcriptome sequencing data from the Gene Expression Omnibus database (GSE266883). Differentially expressed mRNAs were visualized using a heatmap and a volcano plot (Figure 1N,O). Previous studies have demonstrated that excessive mechanical stress can induce cytoskeletal disorganization and degeneration of NP cells. We hypothesized that cytoskeletal proteins played an important role in IDD induced by excessive mechanical stress. We screened the top 40 cytoskeletal genes (relevance score > 10) involved in mechanotransduction and sensitive to mechanical pressure from the GeneCards database (Figure 1P). Intersecting these with the differentially expressed mRNAs from GSE266883 yielded three differentially expressed genes (Figure 1Q,R). The qRT‐PCR results showed that Vimentin's mRNA expression was significantly reduced in human NP tissues with grade V (Figure 1S).

We further validated the bioinformatics results. Immunohistochemistry showed that compared with grade II NP tissue, the expression level of Vimentin was significantly decreased in grade V (Figure 1T,U). Immunofluorescence demonstrated that the number of Vimentin‐positive cells was reduced in the TCS group (Figure 1V,W). Western blot revealed significantly reduced Vimentin expression in compressed NP cells (Figure 1X,Y). Moreover, the morphology of NP cells changed from spindle‐shaped to polygonal or round under compression (Figure S1K). It has been reported that pyroptosis is characterized by cell swelling into a round shape followed by rupture [9]. We hypothesized that the downregulation of Vimentin may be associated with excessive mechanical stress‐induced pyroptosis of NP cells and IDD.

### 3.2 Vimentin Deficiency Accelerates NP Cell Pyroptosis and Coccygeal IDD Induced by TCS in Rats

**FIGURE 2:**
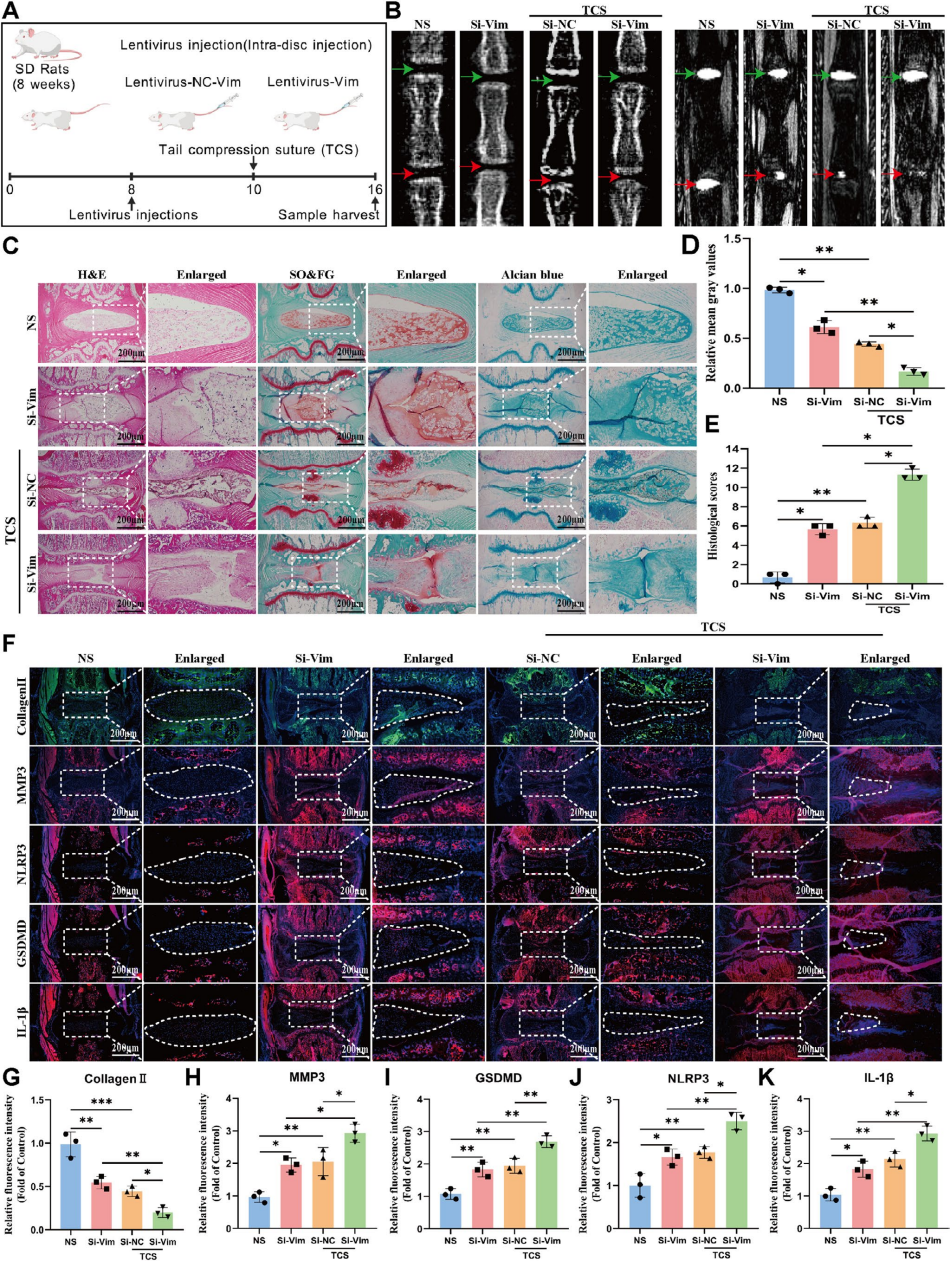
Vimentin deficiency accelerates NP cell pyroptosis and coccygeal IDD induced by TCS in rats. (A) Schematic diagram explaining the workflow and the group settings of animal experiments. (B) Representative images of X‐ray and MRI at the T2‐weighted sequence of coccygeal IVDs in rats, the green arrows indicate the control disc, and the red arrows indicate the surgical disc. (C, E) H&E, SO&FG, and Alcian blue staining of rat coccygeal IVDs in different groups (*n* = 3). Scale bar: 200 μm. (D) Quantitative analysis of NP water content in each group (*n* = 3). (F–K) Immunofluorescence staining of Collagen II, MMP3, NLRP3, GSDMD, and IL‐1β in NP tissues of rat coccygeal IVDs from each group (*n* = 3). Scale bar: 200 μm. Data are represented as mean ± SD. *p* value was calculated with ANOVA. *p < 0.05, **p < 0.01, ***p < 0.001.

Next, we investigated the role of Vimentin deficiency in excessive mechanical stress‐induced pyroptosis and IDD. As shown in Figure 2A, we established a TCS‐induced rat IDD model following lentivirus‐mediated knockdown of Vimentin expression. IF staining indicated that Vimentin was expressed at low levels in NP cells of the coccygeal IVD in the Si‐Vim rats (Figure S2A,B). X‐ray and MRI revealed that Vimentin knockdown significantly reduced DHI and NP water content in rats and accelerated the loss of NP water content in TCS rats (Figure 2B,D). H&E and SO&FG staining demonstrated that rats with knockdown of Vimentin exhibited characteristic degenerative changes compared with the saline group, including a diminished NP area, disorganized annulus fibrosus (AF) lamellae, and loss of the distinct boundary between the NP and AF. More severe degenerative features were observed in TCS rats with Vimentin knockdown (Figure 2C,E). Immunofluorescence staining showed that Vimentin knockdown reduced the expression of collagen II while increasing the expression of MMP3 and exacerbated the ECM metabolic imbalance induced by TCS (Figure 2F–H). Immunofluorescence staining revealed that Vimentin knockdown significantly upregulated the levels of NLRP3, GSDMD, and IL‐1β in the rat NP compared with the control group (saline) and exacerbated the upregulation of NLRP3, GSDMD, and IL‐1β levels in the rat NP induced by TCS (Figure 2F,I–K). These results demonstrate that Vimentin knockdown accelerates the progression of NP cell pyroptosis and IDD caused by excessive mechanical stress in rats.

### 3.3 Overexpression of Vimentin Alleviates NP Cell Pyroptosis and ECM Metabolic Imbalance Under Compression

**FIGURE 3:**
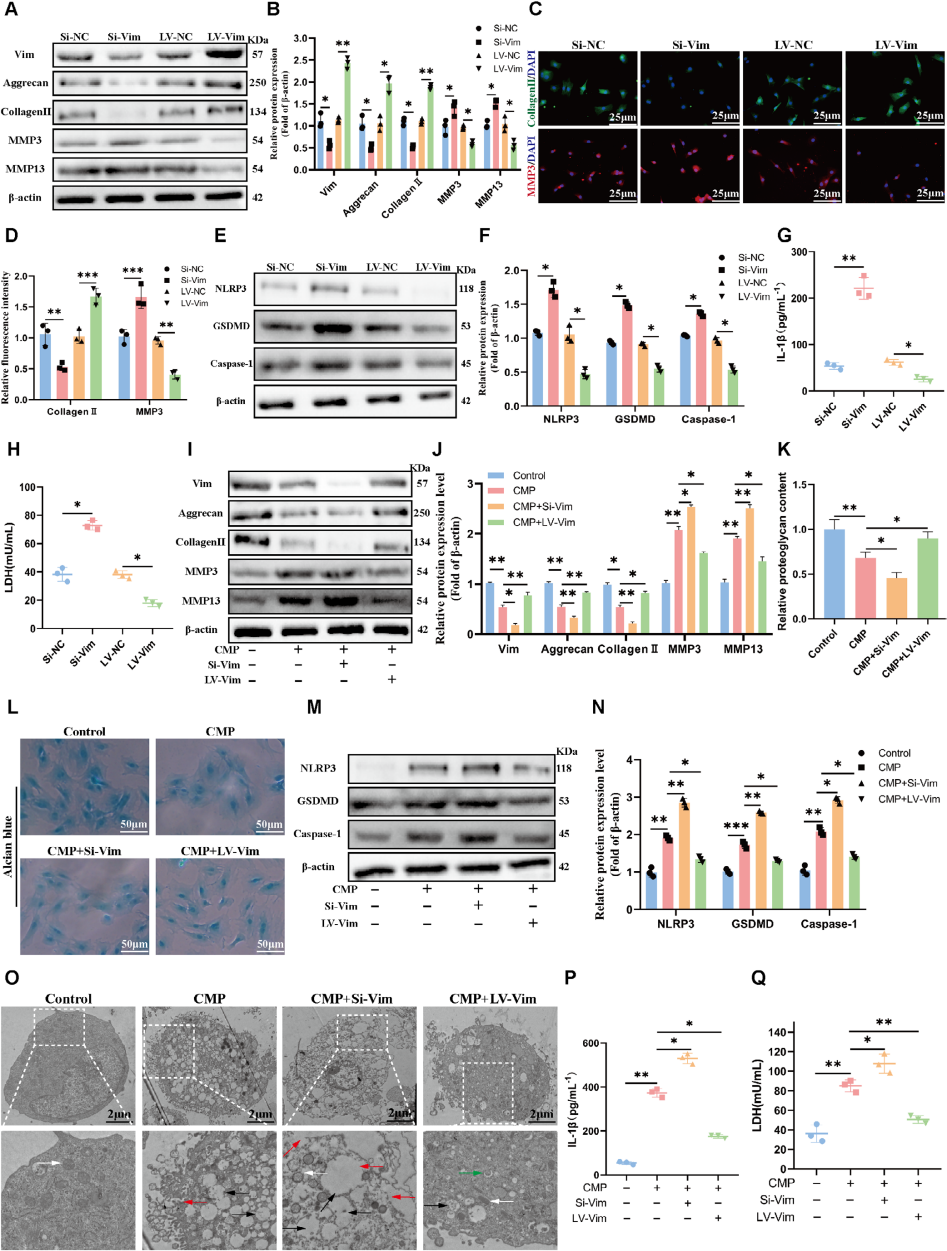
Overexpression of Vimentin alleviates NP cell pyroptosis and ECM metabolic imbalance under compression. (A, B) The protein expression in NP cell with Vimentin knockdown or overexpression was detected by western blotting (*n* = 3). (C, D) Immunofluorescence staining of Collagen II, MMP3 in NP cell in each group (*n* = 3). Scale bar: 25 μm. (E, F) The protein expression in NP cell in each group was determined by western blotting (*n* = 3). (G) The Level of IL‐1β in NP cell in each group was detected by an ELISA kit (*n* = 3). (H) The level of LDH in NP cell of each group was determined by LDH assay (*n* = 3). (I, J) The protein expression in NP cell treated by compression with Vimentin knockdown or overexpression, as determined by western blotting (*n* = 3). (K, L) The proteoglycan content in NP cell of each group was detected by Alcian blue staining (*n* = 3). Scale bar: 50 μm. (M, N) The protein expression in NP cell of each group was determined by western blotting (*n* = 3). (O) Representative transmission electron microscopy images of NP cell in each group. The white arrow points to the mitochondria, the black arrow points to the pyroptotic bodies, the red arrow points to the broken cell membrane, and the green arrow points to the mitochondrial autophagosomes. (P) The level of IL‐1β in NP cell of each group was detected by an ELISA kit (*n* = 3). (Q) The level of LDH in NP cell of each group was determined by LDH assay (*n* = 3). Data are represented as mean ± SD. *p* value was calculated with ANOVA. *p < 0.05, **p < 0.01, ***p < 0.001.

To further explore the role of Vimentin in NP cell pyroptosis and ECM metabolism, we knocked down and overexpressed the level of Vimentin in rat primary NP cells using lentivirus. Knockdown and overexpression efficiencies were confirmed by qRT‐PCR and Western blot (Figure S2C–F). Vimentin knockdown reduced the expression levels of Aggrecan and Collagen II, while increasing the expression of MMP3 and MMP13 (Figure 3A–D). Similarly, Vimentin knockdown reduced the proteoglycan content in NP cells (Figure S2G,H). Vimentin knockdown upregulated the expression of NLRP3, GSDMD, and Caspase‐1 (Figure 3E,F). ELISA and LDH assays confirmed that Vimentin knockdown increased the secretion of IL‐1β and LDH (Figure 3G,H). Moreover, Vimentin overexpression alleviated compression‐induced catabolism in NP cells (Figure 3I,J). Alcian blue staining demonstrated that Vimentin overexpression mitigated the compression‐induced loss of proteoglycans in NP cells (Figure 3K,L). Vimentin overexpression reduced the compression‐induced upregulation of NLRP3, GSDMD, and Caspase‐1 protein levels (Figure 3M,N). Transmission electron microscopy revealed that compression led to an increase in inflammasomes and loss of plasma membrane integrity in NP cells, and Vimentin overexpression attenuated it (Figure 3O). ELISA and LDH assays showed that Vimentin overexpression mitigated the compression‐induced release of IL‐1β and LDH from NP cells (Figure 3P,Q). Collectively, these results suggested that Vimentin alleviates compression‐induced NP cell pyroptosis and ECM metabolic imbalance.

### 3.4 Vimentin Alleviates NP Cell Pyroptosis and ECM Metabolic Imbalance Under Compression by Improving Mitophagy

**FIGURE 4:**
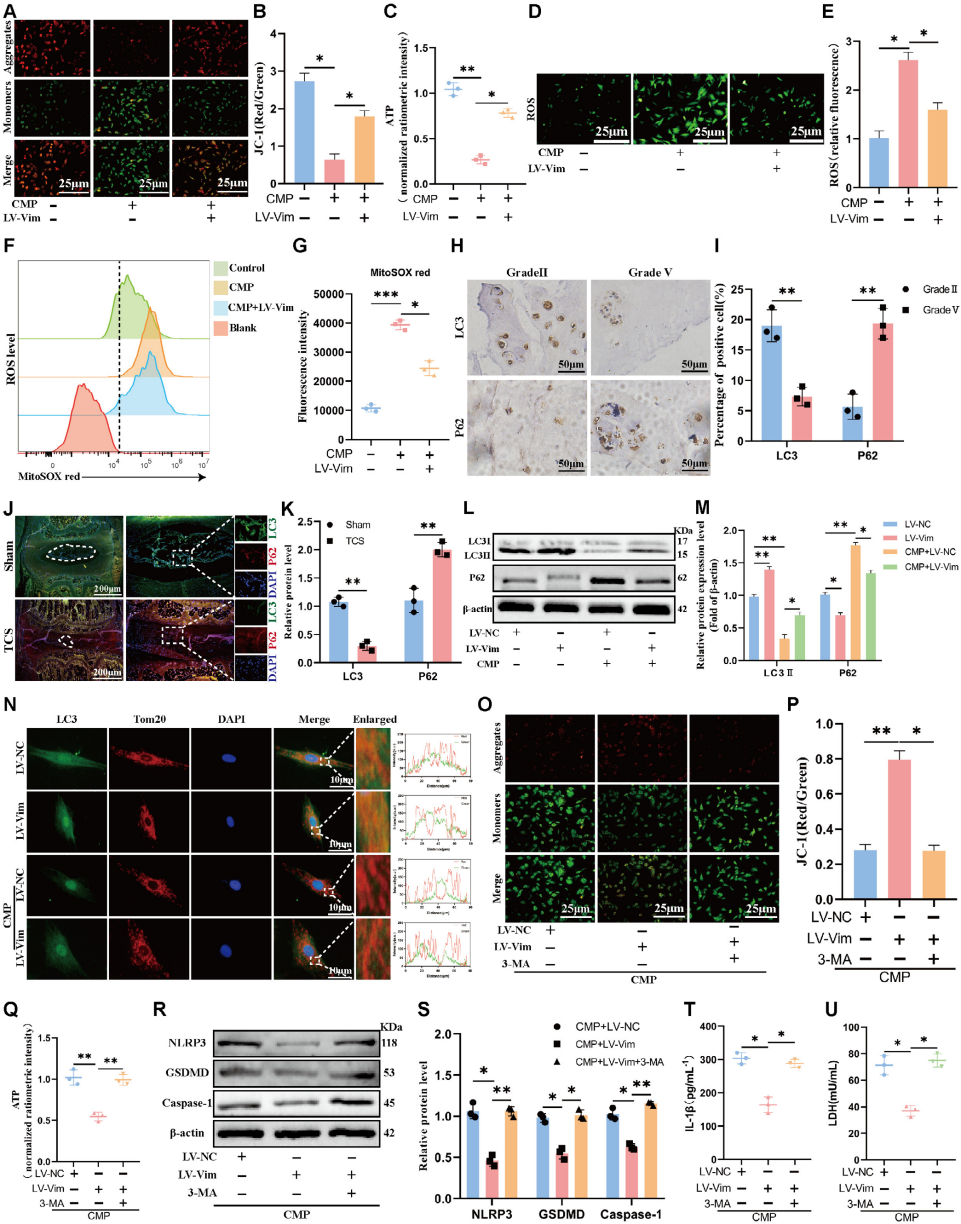
Vimentin alleviates NP cell pyroptosis and ECM metabolic imbalance under compression by improving mitophagy. (A, B) The JC‐1 fluorescent probe was employed to analyse mitochondrial membrane potential in NP cell (*n* = 3). Scale bar: 25 μm. (C) Detection of ATP content in NP cell of each group (*n* = 3). (D, E) DCFH‐DA was utilized to detect total cellular ROS (*n* = 3). Scale bar: 25 μm. (F, G) MitoSOX red was utilized to detect mitochondrial ROS in NP cell (*n* = 3). (H, I) Immunohistochemical staining of LC3 and P62 in human NP tissues with different degrees of degeneration (*n* = 3). Scale bar: 50 μm. (J, K) Immunofluorescence staining of LC3 and P62 in rat coccygeal IVDs (*n* = 3). Scale bar: 200 μm. (L, M) The protein expression of LC3 and P62 in NP cell of each group was determined by western blotting (*n* = 3). (N) The colocalization of LC3 and Tom20 was examined by immunofluorescence colocalization staining. Scale bar: 10 μm. (O, P) The JC‐1 fluorescent probe was employed to analyse mitochondrial membrane potential in NP cell treated by Vimentin overexpression or 3‐MA (*n* = 3). Scale bar: 25 μm. (Q) Detection of ATP content in NP cell of each group (*n* = 3). (R, S) The protein expression in NP cell of each group was determined by western blotting (*n* = 3). (T) The IL‐1β level in NP cell of each group was detected by an ELISA kit (*n* = 3). (U) The level of LDH in NP cell of each group was determined by LDH assay (*n* = 3). Data are represented as mean ± SD. *p* value was calculated with ANOVA. *p < 0.05, **p < 0.01, ***p < 0.001.

The deficiency of Vimentin can impair the morphology and function of mitochondria [21]. Mitochondrial dysfunction induces the production of ROS and triggers pyroptosis by activating the NLRP3 inflammasome [29]. To elucidate the mechanism by which Vimentin ameliorates the pyroptosis and ECM metabolic imbalance of NP cell induced by compression, we evaluated the effect of Vimentin on mitochondrial function in NP cells. The MMP and ATP content of NP cells were decreased under compression, and overexpression of Vimentin attenuated compression‐induced loss of MMP and ATP content (Figure 4A–C). Furthermore, we observed that compression increased ROS levels in NP cells; overexpression of Vimentin could attenuate compression‐induced ROS increase (Figure 4D,E). Similarly, the level of mitochondrial superoxide in NP cells was increased under compression, and overexpression of Vimentin reduced the increase in mitochondrial superoxide induced by compression (Figure 4F,G). These results demonstrate that Vimentin improves mitochondrial function and reduces ROS levels in NP cells under compression.

Mitophagy plays a critical role in maintaining mitochondrial homeostasis and cellular survival [30, 31]. We found that compared with grade II NP tissue, the expression of LC3 was reduced and the accumulation of p62 protein was increased in grade V (Figure 4H,I). The expression of LC3 was decreased, and the expression of p62 was increased in TCS‐induced degenerated rat NP tissue (Figure 4J,K). Moreover, western blot analysis showed that overexpression of Vimentin attenuated the decreased LC3‐II/I ratio and increased p62 expression in compressed NP cells (Figure 4L,M). Immunofluorescence staining demonstrated that overexpression of Vimentin increased the expression of LC3 on mitochondria under compression (Figure 4N). These results indicate that Vimentin ameliorates mitophagy impairment induced by compression.

Next, we treated Vimentin‐overexpressing NP cells with 3‐methyladenine (3‐MA) to block mitophagy. The protective effect of Vimentin overexpression against compression‐induced mitochondrial damage (loss of MMP and reduction in ATP) was abrogated by 3‐MA (Figure 4O–Q). The results indicated that the protective effect of Vimentin overexpression in alleviating compression‐induced pyroptosis of NP cells was abolished by 3‐MA (Figure 4R–U). Moreover, the effect of Vimentin overexpression on ameliorating compression‐induced extracellular matrix degradation was also reversed by 3‐MA (Figure S2I–L). Collectively, these results demonstrate that Vimentin alleviates compression‐induced NP cell pyroptosis and ECM metabolic imbalance by improving mitophagy.

### 3.5 Vimentin Alleviates NP Cell Pyroptosis and ECM Metabolic Imbalance Under Compression Through PINK1‐Parkin‐Dependent Mitophagy

**FIGURE 5:**
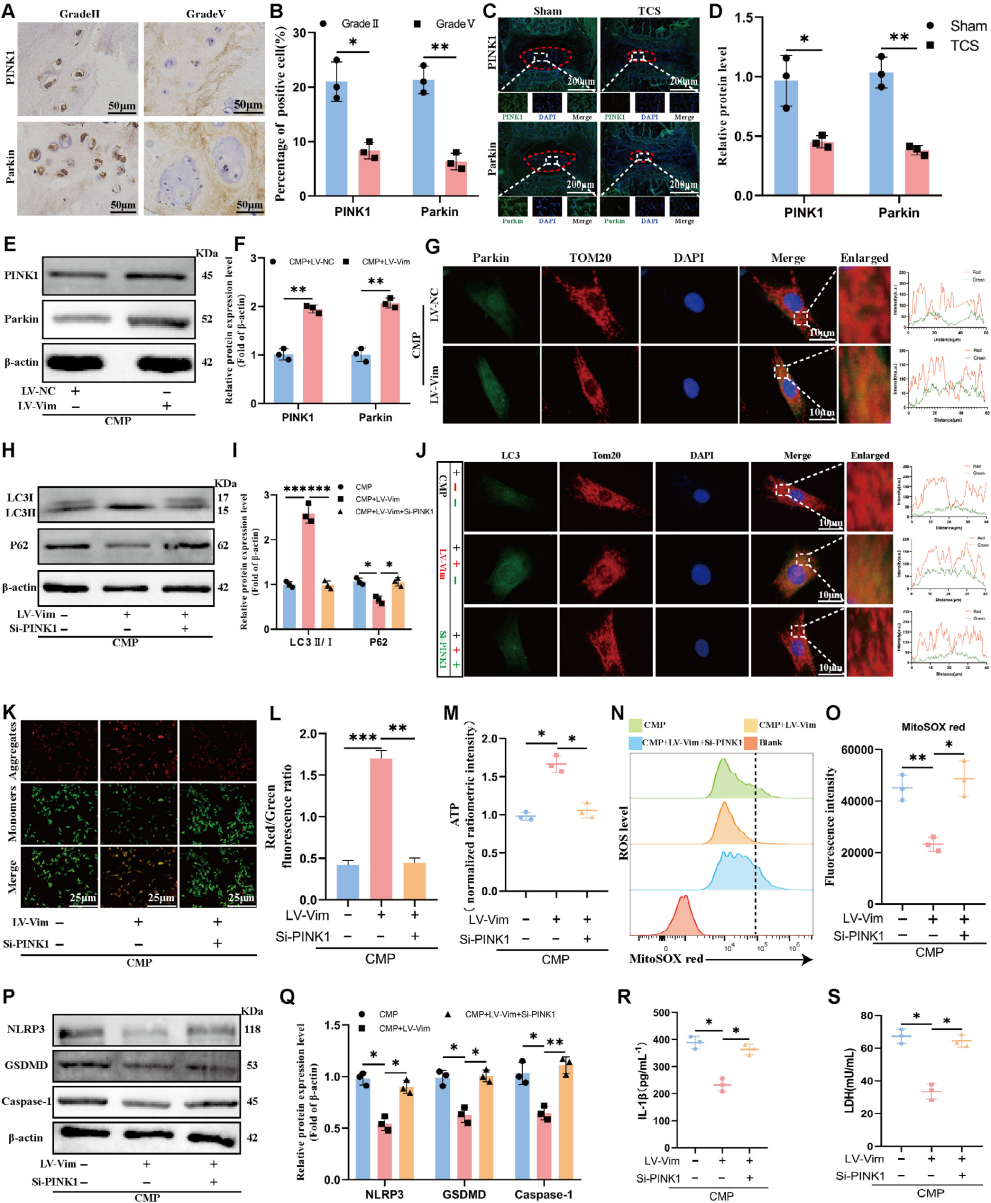
Vimentin alleviates NP cell pyroptosis and ECM metabolic imbalance under compression through PINK1‐Parkin‐dependent mitophagy. (A, B) Immunohistochemical staining of PINK1 and Parkin in different degenerative human NP tissues (*n* = 3). Scale bar: 50 μm. (C, D) Immunofluorescence staining of PINK1 and Parkin in coccygeal IVDs of rats (*n* = 3). Scale bar: 200 μm. (E, F) The protein expressions of PINK1 and Parkin in NP cell of each group were determined by western blotting (*n* = 3). (G) The colocalization of Parkin and Tom20 was examined by immunofluorescence colocalization staining. Scale bar: 10 μm. (H, I) The protein expressions of LC3 and P62 in NP cell of each group were determined by western blotting (*n* = 3). (J) The colocalization of LC3 and Tom20 were examined by immunofluorescence colocalization staining. Scale bar: 10 μm. (K, L) The JC‐1 fluorescent probe was employed to analyse mitochondrial membrane potential in NP cell (*n* = 3). Scale bar: 25 μm. (M) Detection of ATP content in NP cell of each group (*n* = 3). (N, O) MitoSOX red was utilized to detect mitochondrial ROS in NP cell (*n* = 3). (P, Q) The protein expression in NP cell of each group was determined by western blotting (*n* = 3). (R) The levels of IL‐1β in NP cell of each group was detected by an ELISA kit (*n* = 3). (S) The level of LDH in NP cell of each group was determined by LDH assay (*n* = 3). Data are represented as mean ± SD. *p* value was calculated with t‐test or ANOVA. *p < 0.05, **p < 0.01, ***p < 0.001.

The PINK1‐Parkin signalling pathway represents the most common mechanism regulating mitophagy [32, 33]. We found that the expression of PINK1 and Parkin was decreased in human NP tissue with grade V compared with grade II (Figure 5A,B). Similarly, TCS‐induced degenerated NP tissue exhibited decreased expression of PINK1 and Parkin compared with the sham group (Figure 5C,D). Furthermore, the expression of PINK1 and Parkin was decreased in compressed NP cells, while overexpression of Vimentin increased the expression of PINK1 and Parkin under compression (Figure 5E,F). Immunofluorescence co‐localization analysis showed that overexpression of Vimentin increased the co‐localization of Parkin and Tom20 in compressed NP cells (Figure 5G).

To further elucidate whether Vimentin influences NP cell mitophagy via the PINK1‐Parkin pathway, we knocked down PINK1 expression using siRNA. The efficiency of PINK1 knockdown at both the mRNA and protein levels was confirmed by qRT‐PCR and Western blot, respectively (Figure S3A,B). Vimentin overexpression increased the LC3‐II/I ratio and decreased p62 expression in NP cells under compression, which was eliminated by knockdown of PINK1 (Figure 5H,I). Immunofluorescence co‐localization analysis showed that the increase in mitochondrial LC3 puncta induced by Vimentin overexpression was abolished upon PINK1 knockdown (Figure 5J). Moreover, JC‐1 staining revealed that PINK1 knockdown reversed the protective effect of Vimentin on MMP (Figure 5K,L). PINK1 knockdown reduced ATP content in NP cells (Figure 5M). PINK1 knockdown increased mitochondrial superoxide levels within NP cells (Figure 5N,O). The inhibitory effect of Vimentin on pyroptosis‐associated proteins was reversed by PINK1 knockdown (Figure 5P,Q). The levels of IL‐1β and LDH in NP cells were increased upon PINK1 knockdown (Figure 5R,S). Western blot analysis indicated that PINK1 knockdown reversed the promotive effect of Vimentin on the expression of anabolic ECM components in NP cells (Figure S3C,D). Alcian blue staining showed that PINK1 knockdown abolished Vimentin's ability to mitigate compression‐induced proteoglycan loss (Figure S3E,F).

The above results indicate that overexpression of Vimentin alleviates NP cell pyroptosis and ECM metabolic imbalance induced by compression through PINK1‐Parkin‐dependent mitophagy.

### 3.6 Itgb1 Inhibits the Ubiquitin‐Proteasome Degradation of Vimentin Through MNAT1

**FIGURE 6:**
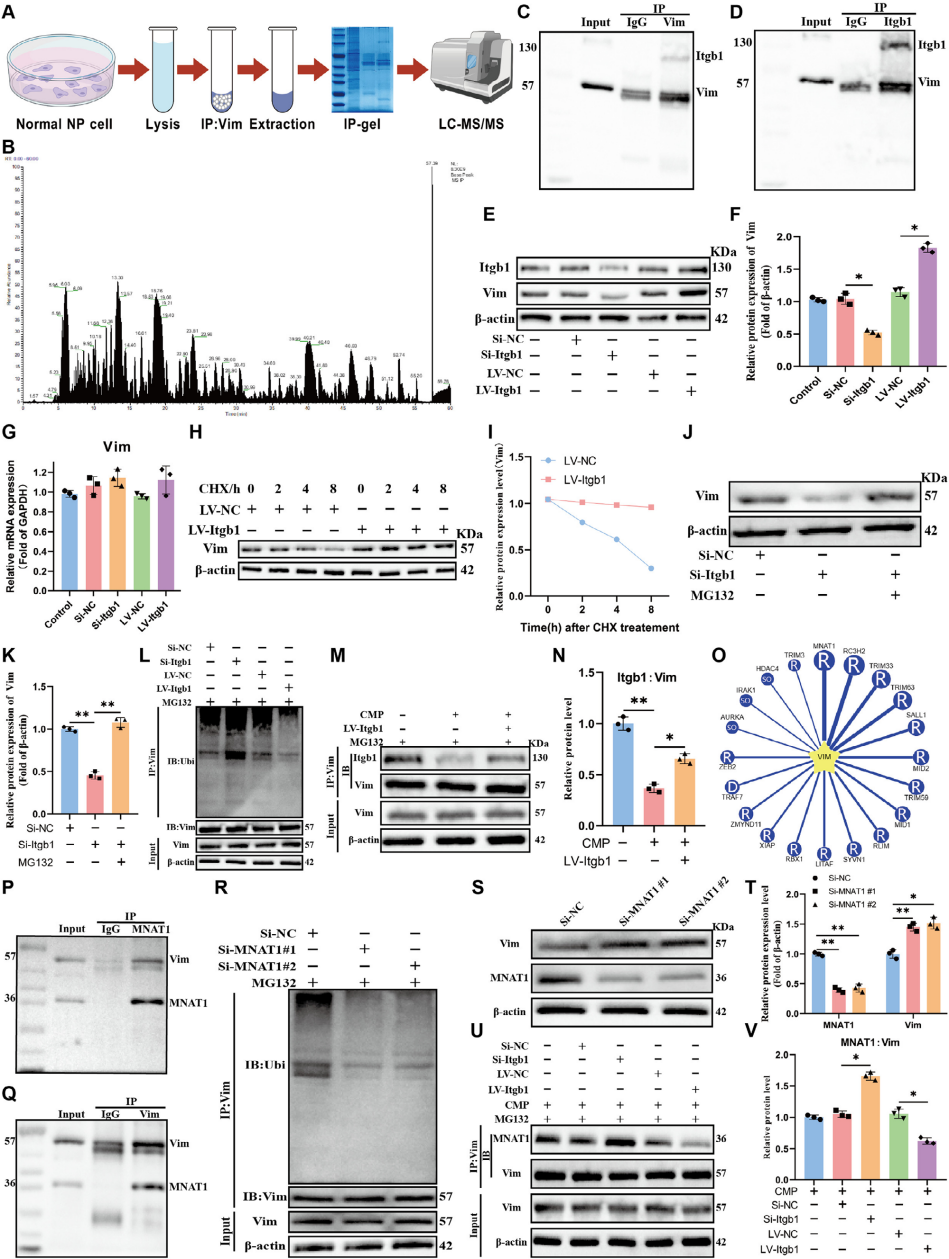
Itgb1 inhibits the ubiquitin‐proteasome degradation of Vimentin through MNAT1. (A, B) The proteins that potentially interact with Vimentin in NP cell were immunoprecipitated with anti‐Vimentin antibody and analysed by mass spectrometry. Endogenous protein immunoprecipitated (IP) from NP cell, followed by western blotting, IP with anti‐Vimentin antibody (C), and IP with anti‐Itgb1 antibody (D). (E, F) The protein expression of Vimentin in NP cell of each group was determined by western blotting (*n* = 3). (G) The mRNA of Vimentin in NP cell with Itgb1 knockdown or Itgb1 overexpression was analysed by qRT‐PCR (*n* = 3). (H, I) The level of Vimentin in NP cell treated with protein synthesis inhibitor CHX (100 μg/mL) was measured (*n* = 3). (J, K) The effect of MG132 treatment on Vimentin protein level alteration mediated by Itgb1 knockdown is shown (*n* = 3). (L) IP analysis of ubiquitinated Vimentin in NP cell treated with MG132. The lysates of Itgb1 overexpression or knockdown cells were treated with an anti‐Vimentin antibody. (M, N) The effect of overexpression of Itgb1 on the binding levels of Itgb1 and Vimentin in compressed NP cell through IP analysis. IP with anti‐Vimentin antibody (*n* = 3). (O) Prediction of potential proteins regulating Vimentin ubiquitination. Endogenous protein IP from NP cell followed by western blotting, IP with anti‐MNAT1 antibody (P), and IP with anti‐Vimentin antibody (Q). (R) Effect of MNAT1 knockdown on Vimentin ubiquitination. (S, T) The protein expression of MNAT1 and Vimentin in NP cell was determined by western blotting (*n* = 3). (U, V) Effect of Itgb1 overexpression or knockdown on the interaction between Vimentin and MNAT1, IP with anti‐Vimentin antibody (*n* = 3). Data are represented as mean ± SD. *p* value was calculated with ANOVA. *p < 0.05, **p < 0.01.

To elucidate the molecular mechanism of downregulation of Vimentin in IDD, we employed immunoprecipitation‐mass spectrometry (IP‐MS) to identify proteins interacting with Vimentin (Figure 6A,B). IP‐MS results indicated that Integrin beta‐1 (Itgb1) was one of the interacting proteins (Figure S3G). Integrins are established transmembrane receptors for cell adhesion, regulating the ECM and cytoskeleton linkage, and participating in mechanotransduction [34]. Immunofluorescence colocalization revealed an interaction between Itgb1 and Vimentin (Figure S3H). Co‐immunoprecipitation (Co‐IP) further confirmed that endogenous Itgb1 could be successfully immunoprecipitated by an anti‐Vimentin antibody, and vice versa (Figure 6C,D). Transcriptome sequencing data from GSE266883 revealed that the expression level of Vimentin was positively correlated with Itgb1 (Figure S3I). Immunohistochemical staining showed decreased Itgb1 expression in human NP tissue with grade V compared with grade II (Figure S3J,K). Moreover, we knocked down and overexpressed Itgb1 expression using lentivirus (Figure S3L–O). Western blot and qRT‐PCR analysis demonstrated that Itgb1 regulates Vimentin protein levels without affecting its mRNA levels (Figure 6E–G). We inhibited new protein synthesis using cycloheximide to investigate the mechanism by which Itgb1 upregulates Vimentin expression. We found that the half‐life of Vimentin was prolonged upon Itgb1 overexpression (Figure 6H,I). MG132 (a proteasome inhibitor) treatment rescued the decline in Vimentin caused by Itgb1 knockdown (Figure 6J,K). Itgb1 knockdown increased Vimentin ubiquitination in NP cells, whereas Itgb1 overexpression decreased it (Figure 6L). These results indicate that Itgb1 upregulates Vimentin levels by suppressing its degradation via the ubiquitin‐proteasome pathway. Additionally Co‐IP analysis revealed that the endogenous interaction between Itgb1 and Vimentin was reduced under compression, and overexpression of Itgb1 reversed this compression‐induced reduction in binding (Figure 6M,N).

We further explored the mechanism by which Itgb1 regulates Vimentin ubiquitination. We predicted potential proteins that regulate Vimentin ubiquitination using the UbiBrowser 2.0 database (Figure 6O). MNAT1 was selected based on a prediction score exceeding 0.8. Immunofluorescence colocalization revealed an interaction between MNAT1 and Vimentin (Figure S4A). Co‐IP further confirmed that endogenous MNAT1 could be successfully immunoprecipitated by an anti‐Vimentin antibody, and vice versa (Figure 6P,Q). MNAT1 knockdown reduced the level of Vimentin ubiquitination (Figure 6R) and increased Vimentin protein expression in NP cells (Figure 6S,T). Furthermore Co‐IP analysis revealed that the endogenous interaction between MNAT1 and Vimentin was increased under compression (Figure S4B,C). Itgb1 knockdown increased the binding between Vimentin and MNAT1 in NP cells under compression. Conversely, Itgb1 overexpression induced the opposite effect (Figure 6U,V). These results suggested that Itgb1 inhibits the degradation of Vimentin by suppressing the binding of MNAT1 to Vimentin. These results highlight the role of Itgb1 in maintaining Vimentin stability.

### 3.7 Itgb1 Alleviates NP Cell Mitophagy Impairment, Pyroptosis, and ECM Metabolic Imbalance Under Compression via Vimentin

**FIGURE 7:**
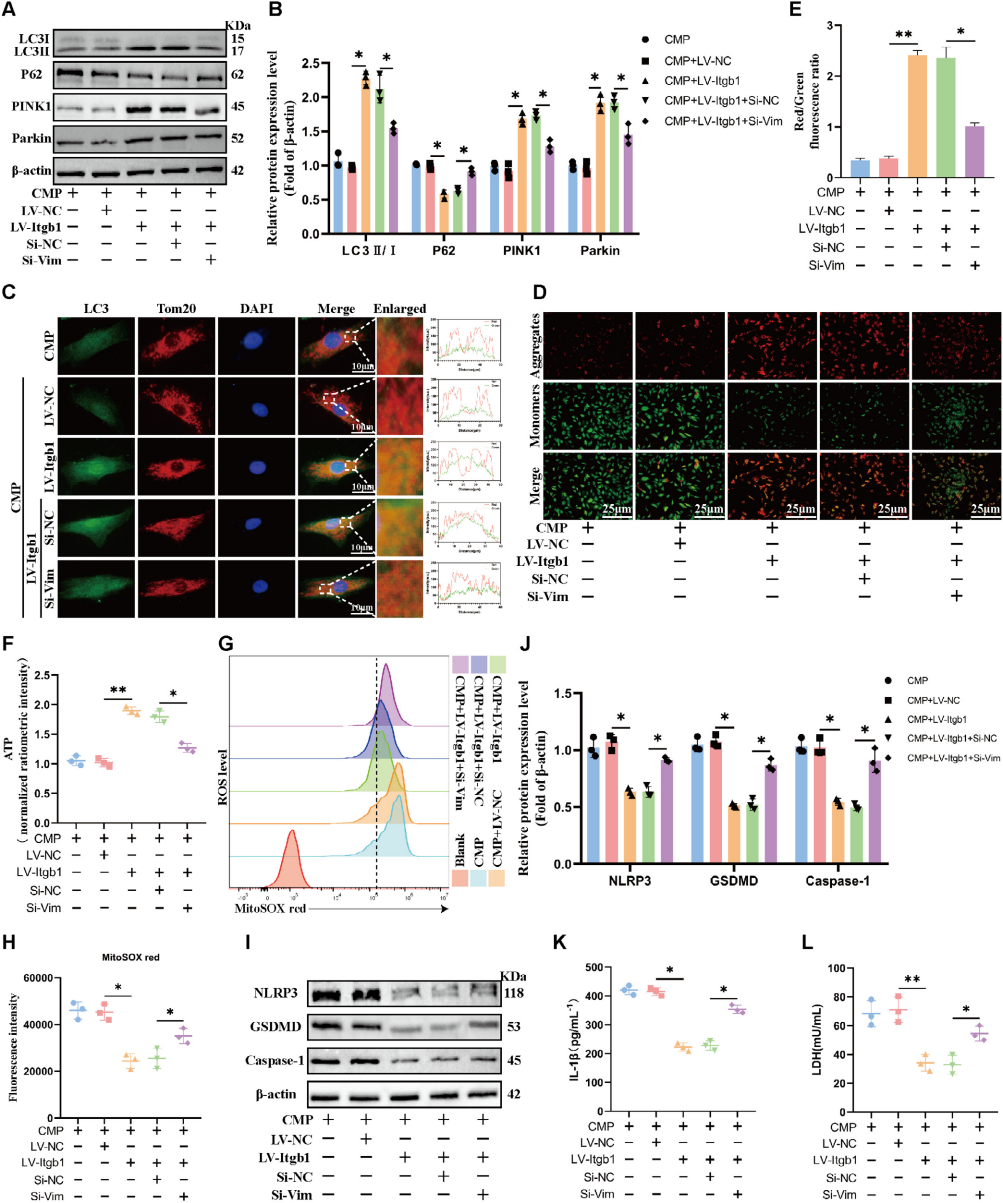
Itgb1 alleviates NP cell mitophagy impairment, pyroptosis, and ECM metabolic imbalance under compression via Vimentin. (A, B) The protein expression in NP cell of each group was determined by western blotting (*n* = 3). (C) The colocalization of LC3 and Tom20 was examined by immunofluorescence colocalization staining. Scale bar: 10 μm. (D, E) The JC‐1 fluorescent probe was employed to analyse mitochondrial membrane potential in NP cell (*n* = 3). Scale bar: 25 μm. (F) Detection of ATP content in NP cell of each group (*n* = 3). (G, H) MitoSOX red was utilized to detect mitochondrial ROS in NP cell (*n* = 3). (I, J) The protein expression in NP cell of each group was determined by western blotting (*n* = 3). (K) The level of IL‐1β in NP cell of each group was detected by an ELISA kit (*n* = 3). (L) The level of LDH in NP cell of each group was detected by LDH assay (*n* = 3). Data are represented as mean ± SD. *p* value was calculated with ANOVA. *p < 0.05, **p < 0.01.

To confirm whether Itgb1 mediates compression‐induced NP cell pyroptosis and ECM metabolic imbalance through Vimentin, we subjected NP cells overexpressing Itgb1 and concurrently knocking down Vimentin to mechanical compression. Overexpressing Itgb1 alleviated the compression‐induced decrease in the LC3‐II/I ratio and increased p62 expression. Vimentin knockdown abrogated this protective effect of Itgb1 (Figure 7A,B). Overexpressing Itgb1 ameliorated the compression‐induced reduction in mitochondrial LC3 puncta; the effect was abolished upon Vimentin knockdown (Figure 7C). JC‐1 staining and ATP assays showed that overexpressing Itgb1 significantly improved MMP and ATP content in compressed NP cells compared with NP cells overexpressing Itgb1 with Vimentin knockdown (Figure 7D–F). Overexpressing Itgb1 significantly reduced mitochondrial superoxide levels in NP cells under compression, while knockdown of Vimentin reversed this effect (Figure 7G,H). Western blot analysis indicated that overexpressing Itgb1 reversed the compression‐induced upregulation of NLRP3, GSDMD, and Caspase‐1; this reversal was counteracted by Vimentin knockdown (Figure 7I,J). Overexpressing Itgb1 reduced the level of IL‐1β and LDH in compressed NP cells, whereas Vimentin knockdown eliminated this effect (Figure 7K,L). In addition, overexpressing Itgb1 enhanced anabolic and reduced catabolic activities in NP cells under compression. This effect was reversed by Vimentin knockdown (Figure S4D,E). Alcian blue staining showed that Vimentin knockdown abolished the ability of overexpressing Itgb1 to mitigate compression‐induced proteoglycan loss (Figure S4F,G). Collectively, Itgb1 alleviates NP cell mitophagy impairment, pyroptosis, and ECM metabolic imbalance via Vimentin under compression.

### 3.8 Vimentin Alleviates NP Cell Pyroptosis and Coccygeal IDD Induced by TCS in Rats

**FIGURE 8:**
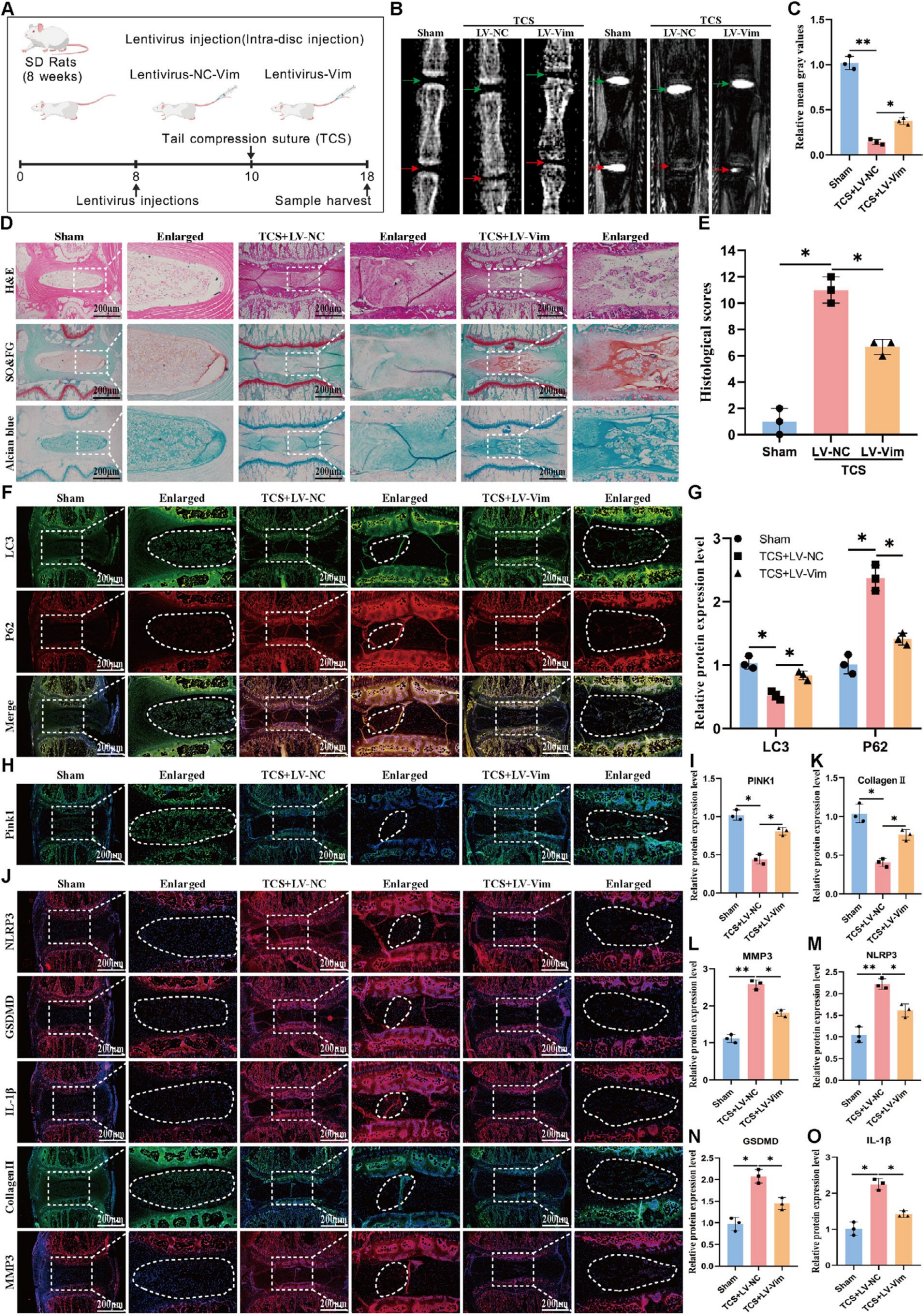
Vimentin alleviates NP cell pyroptosis and coccygeal IDD induced by TCS in rats. (A) Schematic diagram explaining the workflow and the group settings of animal experiments. (B) Representative images of X‐ray and MRI at the T2‐weighted sequence of coccygeal IVDs in rats, the green arrows indicate the control disc, and the red arrows indicate the surgical disc. (C) Quantitative analysis of NP water content in each group (*n* = 3). (D, E) H&E, SO&FG, and Alcian blue staining of rat coccygeal IVDs in each group (*n* = 3). Scale bar: 200 μm. (F, G) Immunofluorescence staining of LC3 and P62 in rat coccygeal IVDs of each group (*n* = 3). Scale bar: 200 μm. (H–I) Immunofluorescence staining of PINK1 in rat coccygeal IVDs of each group (*n* = 3). Scale bar: 200 μm. (J–O) Immunofluorescence staining of Collagen II, MMP3, NLRP3, GSDMD, and IL‐1β in rat coccygeal IVDs of each group (*n* = 3). Scale bar: 200 μm. Data are represented as mean ± SD. *p* value was calculated with ANOVA. *p < 0.05, **p < 0.01.

To explore the effect of Vimentin on IDD induced by excessive mechanical stress in rats, we introduced the lentivirus expressing Vimentin into the IVD through intradiscal injection (Figure 8A). IF staining revealed that the expression level of Vimentin was significantly increased in the NP cells of the coccygeal IVD in the LV‐Vim rats (Figure S4H,I). X‐ray revealed that Vimentin overexpression did not attenuate the TCS‐induced reduction in DHI (Figure 8B). T2‐weighted MRI showed that the NP signal intensity in the LV‐Vim‐treated group was significantly higher than that in the TCS group (Figure 8B,C). H&E and SO&FG staining showed disordered tissue structure in TCS rats; overexpression of Vimentin significantly ameliorated these pathological alterations (Figure 8D,E). Alcian blue staining indicated that overexpressing Vimentin increased proteoglycan synthesis in NP cells under TCS (Figure 8D). Moreover, compared with the TCS group, the expression of p62 was decreased in NP tissue of rats overexpressing Vimentin, while the expression of LC3 and PINK1 was increased (Figure 8F–I). Overexpressing Vimentin increased Collagen II expression and decreased MMP3 expression, while decreasing the expression of NLRP3, GSDMD, and IL‐1β (Figure 8J–O). These results demonstrated that Vimentin overexpression conferred significant protection against TCS‐induced NP cell pyroptosis and rat coccygeal IDD.

## 4 Discussion

IDD is a well‐established major cause of low back pain, yet its underlying mechanisms remain incompletely understood. Excessive mechanical stress is an independent pathogenic factor [35]. Clarifying the detailed mechanism of excessive mechanical stress‐induced IDD is crucial for developing treatment strategies targeting potential mechanisms:

In this study, we established a pressure‐induced in vitro degeneration model of NP cells. Based on available evidence, the pressure borne by human IVDs is ~1.1 MPa in the standing forward flexion posture [36]. NP cells are exposed to dynamic mechanical loading under physiological conditions, and mechanical stress of 0.8–1.3 MPa can trigger NP cell death and ECM degradation [37]. We successfully established an NP cell degeneration model via dynamic compression (1 MPa and 1 Hz), which was consistent with previous findings [38].

The study confirmed that the levels of pyroptosis in human NP tissues were positively correlated with the degree of IDD. Moreover, mechanical stress significantly upregulated the pyroptosis level in the rat NP. These findings are consistent with previous results [7]. Collectively, these results further refine the role of pyroptosis in IDD induced by excessive mechanical stress. However, the specific molecular mechanisms by which pyroptosis participates in excessive mechanical stress‐induced IDD remain unclear.

We identified Vimentin as a pivotal gene involved in excessive mechanical stress‐induced IDD via bioinformatics analysis. The dataset GSE266883 used in this study was derived from a lumbar instability model. Although it differs from the TCS model adopted in our study, both models essentially represent degenerative changes triggered by excessive compressive loading. Surgical resection of posterior structures including articular processes and ligaments disrupts the normal load distribution of the spine, resulting in abnormal stress concentration and excessive compressive loading on IVDs. A study has confirmed that lumbar instability can directly elevate intradiscal pressure and induce IDD [39]. We further verified that Vimentin expression was downregulated in TCS‐induced IDD, which was consistent with the bioinformatics prediction results. The identification of Vimentin in these two distinct mechanical models further validates its role as a core mechanosensitive molecule in the pathogenesis of IDD.

Importantly, we demonstrated for the first time the critical role of Vimentin in NP cell pyroptosis and IDD induced by excessive mechanical stress. In vivo experiments showed that knocking down Vimentin significantly reduced DHI and water content in the NP of rat IVDs and increased histological scores. This phenotype closely resembles that observed in human IDD. Cell death and ECM metabolic imbalance are the two main characteristics of human IDD [40, 41]. Vimentin knockdown disrupted ECM metabolic balance and increased the levels of pyroptosis in rat NP cells. Vimentin knockdown accelerated TCS‐induced NP cell pyroptosis and IDD in rats. Moreover, in vitro studies demonstrated that Vimentin overexpression mitigated these effects.

This study demonstrates for the first time that Vimentin inhibits excessive mechanical stress‐induced pyroptosis of NP cells and IDD by enhancing mitophagy. Accumulation of ROS resulting from mitochondrial dysfunction is an important mechanism for activating the NLRP3 inflammasome [42, 43]. Vimentin, a member of the intermediate filament family, colocalizes and directly interacts with mitochondria. Knockdown of Vimentin leads to mitochondrial fragmentation, swelling, and structural disorganization [21]. Vimentin regulates mitochondrial function and ROS levels in neutrophils [44]. We found that MMP declined and ATP content decreased in compressed NP cells, while cellular ROS and mitochondrial superoxide increased. Overexpression of Vimentin effectively reversed these mitochondrial injuries. These results demonstrate that Vimentin protects against mitochondrial damage induced by excessive mechanical stress. Mitophagy maintains mitochondrial homeostasis by clearing damaged mitochondria. Numerous studies have reported that mitophagy dysfunction is involved in the progression of IDD [45, 46, 47]. However, the role of the cytoskeleton in mitophagy‐mediated IDD remains unclear. Wang et al. [48] found that keratin 8 (an intermediate filament) alleviates mechanical stress‐induced IDD by regulating autophagy. This study revealed that degenerated NP cells exhibited marked mitophagy dysfunction, as evidenced by a decreased LC3‐II/I ratio and accumulation of P62 protein. Overexpression of Vimentin significantly restored mitophagy levels in NP cells following compression. Treatment with 3‐MA significantly blocked the inhibitory effects of Vimentin overexpression on pyroptosis and the amelioration of ECM imbalance in NP cells. Collectively, these findings confirm that under excessive mechanical stress, Vimentin inhibits pyroptosis of NP cells and corrects ECM metabolic dysregulation through mitophagy. These results further strengthen the role of cytoskeletal proteins in IDD.

The PINK1‐Parkin pathway is a key regulator of mitophagy [49, 50]. This study demonstrates that Vimentin alleviates excessive mechanical stress‐induced NP cell pyroptosis through PINK1‐Parkin‐mediated mitophagy. Notably, PINK1 knockdown partially abolished the protective effect of Vimentin, indicating that the PINK1‐Parkin pathway is a critical component of this effect. However, we cannot exclude the possibility that Vimentin may also simultaneously regulate receptor‐mediated pathways (BNIP3, NIX, FUNDC1). Under mechanical stress, BNIP3 and NIX may be mildly activated. Further investigations are required to clarify whether they are modulated by Vimentin or undergo compensatory upregulation upon PINK1 deficiency. Existing studies have demonstrated that cytoskeletal reorganization attenuates Parkin recruitment and leads to mitochondrial damage [51]. Furthermore, Vimentin itself lacks kinase or transcriptional activity, and its regulation of the PINK1‐Parkin pathway is likely achieved through indirect mechanisms. Vimentin serves as a scaffold for multiple kinases (e.g., Akt, PKC, ERK) and the adaptor protein p62, thereby bringing PINK1/Parkin into close proximity with mitochondrial substrates and facilitating mitophagy [52].

This study demonstrates that Itgb1 enhances Vimentin stability by reducing ubiquitination of Vimentin. Knockdown of Itgb1 significantly upregulated the ubiquitination of Vimentin, whereas overexpression of Itgb1 inhibited Vimentin ubiquitination and degradation. As a major post‐translational modification, ubiquitination regulates numerous physiological processes, including chondrocyte metabolism. Li et al. [25] demonstrated that circARPC1B protects against high cholesterol‐induced articular cartilage degeneration by inhibiting Vimentin ubiquitination. Further mechanistic investigations revealed that overexpression of MNAT1 promoted Vimentin ubiquitination, whereas knockdown of MNAT1 inhibited it. Co‐IP assays confirmed that binding of Itgb1 to Vimentin significantly reduced the binding efficiency between MNAT1 and Vimentin. The underlying mechanism may involve identical binding sites for Itgb1 and MNAT1 on Vimentin, or alternatively, Itgb1 binding to Vimentin may induce a conformational change in Vimentin, thereby decreasing its affinity for MNAT1. There may be a competitive relationship between Itgb1 and MNAT1. Future studies using peptide mapping analysis or in vitro competitive binding assays are required to further clarify this mechanism. Collectively, we found that Itgb1 inhibits Vimentin degradation by obstructing the binding of MNAT1 to Vimentin. This provides a potential mechanism to explain the reduced Vimentin expression levels in degenerated NP tissue.

In addition, our study demonstrates that MNAT1 promotes the ubiquitination of Vimentin. However, MNAT1 lacks canonical E3 ubiquitin ligase catalytic domains, such as RING or HECT. MNAT1 may therefore mediate Vimentin ubiquitination through indirect mechanisms. MNAT1 may act as an adaptor protein that recruits a classical E3 ubiquitin ligase to target Vimentin. Such adaptor‐dependent substrate recognition is common in ubiquitination regulatory systems, as exemplified by F‐box proteins [53]. Alternatively, as a component of the CDK‐activating kinase complex, MNAT1 can regulate E3 ligase activity through CDK‐mediated phosphorylation, thereby affecting the function of the ubiquitin‐proteasome system [54]. The current results do not distinguish between these two possibilities. Future in vitro ubiquitination assays are required to determine whether MNAT1 directly catalyses or merely facilitates Vimentin ubiquitination. Nevertheless, our findings demonstrate that under mechanical stress, MNAT1 is functionally important for Vimentin ubiquitination and degradation.

Previous studies have reported the role of the interaction between Itgb1 and Vimentin in various biological behaviours. In hepatocellular carcinoma, the interaction between Itgb1 and Vimentin promotes hepatocellular carcinoma cell motility [55]. The interaction between integrin and Vimentin is involved in the migration and invasion of non‐small cell lung cancer cells [56]. However, the roles of Itgb1 and Vimentin in NP cell degeneration have not been elucidated. We found that Itgb1 alleviated excessive mechanical stress‐induced NP cell pyroptosis and ECM metabolic imbalance via Vimentin.

Our study demonstrated that overexpression of Vimentin ameliorated coccygeal IDD induced by TCS in rats. However, although Vimentin overexpression improved the MRI signal of the NP, it failed to attenuate the reduction in DHI. This phenomenon may be attributed to the sustained compressive force exerted on the IVD by the compression suture. The reduction in DHI induced by persistent suture compression is affected by NP conditions, overall disc structure, and continuous tension of surrounding ligaments. Simple upregulation of Vimentin may fail to fully reverse structural height decline under sustained mechanical stress.

Nonetheless, our study has several limitations that require further refinement. Firstly, we successfully simulated excessive mechanical stress‐induced IDD by constructing a TCS rat model. Although this model shares pathological and histological changes with human IDD, it must be acknowledged that rodent discs differ from those of large mammals in terms of function and biomechanical properties. We plan to include primate models in future studies to mimic human spinal biomechanics more accurately. Secondly, rats retain notochord‐like NP cells throughout most of their lifespan, whereas these cells gradually disappear in humans with age. This species‐specific functional difference may limit our exploration of human IDD. Therefore, we need to address the differences in developmental biology between rats and humans in the future. Thirdly, although our results showed that administering lentivirus expressing Vimentin via direct injection into the IVD alleviated excessive mechanical stress‐induced IDD in rats, the potential toxicity to other organs remains unclear and requires long‐term experimental studies in the future.

## 5 Conclusion

In summary, our study demonstrates that Vimentin alleviates excessive mechanical stress‐induced NP cell pyroptosis and IDD through PINK1‐Parkin‐dependent mitophagy. Itgb1 inhibits the degradation of Vimentin through the ubiquitin‐proteasome pathway. In addition, this study confirmed that Vimentin overexpression can delay excessive mechanical stress‐induced IDD in rats. Therefore, this study enriches the molecular mechanisms of excessive mechanical stress‐induced IDD and provides a potential therapeutic target for IDD.

## Author Contributions

Xuening Liu, Xuewen Kang, and Fengguang Yang conceived and designed the idea for this paper. Yanni Duan and Hefang Xiao collected and analysed the data. Zhenyu Cao participated in the construction of a rat model and the collection of human samples. Xuening Liu contributed to the visualization and the original draft preparation. Xuewen Kang, Zhaoheng Wang, and Haijun Zhang contributed to revising the manuscript. All authors have read and approved the article.

## Funding

This work was supported by the National Natural Science Foundation of China (Grant Nos. 82272536 and 82460436), the Natural Science Foundation of Gansu province (Grant No. 23JRRA1626), and the Cuiying Scientific and Technological Innovation Program of The Second Hospital & Clinical Medical School, Lanzhou University (Grant Nos. CY2022‐ZD‐02 and CY2025‐YB‐A09).

## Ethics Statement

This study was approved by the Ethics Committee of the Second Hospital of Lanzhou University (Approval No. 2025A‐559). All animal experiments were approved by the Animal Ethics Committee of the Second Hospital of Lanzhou University (Approval No. D2025‐427).

## Consent

Written informed consent was obtained from each donor.

## Conflicts of Interest

The authors declare no conflicts of interest.

## Supporting information


**Table S1:** The primary antibodies for IHC, IF, and western blot.
**Figure S1:** (A) H&E, Alcian blue, and Safranin‐O staining of human NP samples. Scale bar: 50 μm. (B, C) Immunohistochemical staining of Collagen II, MMP3, NLRP3, GSDMD, and IL‐1β in different degenerative human NP tissues (*n* = 3). Scale bar: 50 μm. (D, E) The protein expressions of ECM (Collagen II, MMP3) and pyroptosis indicators (NLRP3, GSDMD, and IL‐1β) in different degenerative NP tissues, as determined by western blotting (*n* = 3). (F) Quantitative analysis of intervertebral disc height in rats (*n* = 3). (G) Quantitative analysis of water content of nucleus pulposus in rats (*n* = 3). (H) Cell viability of primary rat NP cells subjected to different time length compression (*n* = 3). (I, J) The protein expression levels of Aggracan, Collagen II, MMP3, and MMP13 in primary rat NP cells after being compressed for different time periods, as determined by Western blotting (*n* = 3). (K) Changes in morphology and cytoskeleton of primary NP cells in rats after compression. Data are represented as mean ± SD. *p* value was calculated with t‐test or ANOVA. *p < 0.05, **p < 0.01, ***p < 0.001.
**Figure S2:** (A, B) IF staining of Vim in coccygeal IVDs in rats (*n* = 3). Scale bar: 200 μm. (C, D) The mRNA expression levels of Vimentin in primary rat NP cells in each group was determined by qRT‐PCR (*n* = 3). (E, F) The protein expression levels of Vimentin in primary rat NP cells in each group was determined by Western blotting. (G, H) The proteoglycan content in NP cells of each group was detected by Alcian blue staining (*n* = 3). Scale bar: 50 μm. (I, J) The protein expression levels of Aggracan, Collagen II, MMP3, and MMP13 in primary rat NP cells determined by Western blotting (*n* = 3). (K, L) The proteoglycan content in NP cells of each group was detected by Alcian blue staining (*n* = 3). Scale bar: 50 μm. Data are represented as mean ± SD. *p* value was calculated with *t*‐test or ANOVA. *p < 0.05, **p < 0.01, ***p < 0.001.
**Figure S3:** (A) The mRNA expression levels of PINK1 in primary rat NP cells in each group was determined by qRT‐PCR (*n* = 3). (B) The protein expression levels of PINK1 in primary rat NP cells in each group was determined by Western blotting. (C, D) The protein expression levels of Aggracan, Collagen II, MMP3, and MMP13 in primary rat NP cells determined by Western blotting (*n* = 3). (E, F) The proteoglycan content in NP cells of each group was detected by Alcian blue staining (*n* = 3). Scale bar: 50 μm. (G) IP‐MS analysis revealed the presence of Itgb1. (H) Immunofluorescence colocalization of Itgb1 and Vimentin. Scale bar: 10 μm. (I) The correlation between Itgb1 and Vimentin expression levels in the GSE266883 dataset. (J, K) Immunohistochemical staining of Itgb1 in different degenerative human NP tissues (*n* = 3). Scale bar: 50 μm. (L, M) The mRNA expression levels of Itgb1 in primary rat NP cells in each group was determined by qRT‐PCR (*n* = 3). (N, O) The protein expression levels of Itgb1 in primary rat NP cells in each group was determined by Western blotting. Data are represented as mean ± SD. *p* value was calculated with t‐test or ANOVA. *p < 0.05, **p < 0.01, ***p < 0.001.
**Figure S4:** (A) Immunofluorescence colocalization of MNAT1 and Vimentin. Scale bar: 10 μm. (B, C) The binding levels of MNAT1 and Vimentin in compressed primary rat NP cells through IP analysis. IP with anti‐Vimentin antibody (*n* = 3). (D, E) The protein expression levels of Aggracan, Collagen II, MMP3, and MMP13 in primary rat NP cells determined by Western blotting (*n* = 3). (F, G) The proteoglycan content in NP cells of each group was detected by Alcian blue staining (*n* = 3). Scale bar: 50 μm. (H, I) IF staining of Vim in coccygeal IVDs in rats (*n* = 3). Scale bar: 200 μm. Data are represented as mean ± SD. *p* value was calculated with t‐test or ANOVA. *p < 0.05, **p < 0.01, ***p < 0.001.

## Data Availability

The data that support the findings of this study are available from the corresponding author upon reasonable request.
