# Supplementary material for: Itgb1‐Mediated Stabilization of Vimentin Alleviates Excessive Mechanical Stress‐Induced Nucleus Pulposus Cell Pyroptosis and Intervertebral Disc Degeneration via PINK1‐Parkin‐Dependent Mitophagy
Source: Cell Prolif. 2026 Jul 14:e70256. Online ahead of print. doi: 10.1111/cpr.70256 (PMC13367296; doi:10.1111/cpr.70256)
Supplement: Supplementary file 1 — Table S1: The primary antibodies for IHC, IF, and western blot. Figure S1: (A) H&E, Alcian blue, and Safranin‐O staining of human NP samples. Scale bar: 50 μm. (B, C) Immunohistochemical staining of Collagen II, MMP3, NLRP3, GSDMD, and IL‐1β in different degenerative human NP tissues (n = 3). Scale bar: 50 μm. (D, E) The protein expressions of ECM (Collagen II, MMP3) and pyroptosis indicators (NLRP3, GSDMD, and IL‐1β) in different degenerative NP tissues, as determined by western blotting (n = 3). (F) Quantitative analysis of intervertebral disc height in rats (n = 3). (G) Quantitative analysis of water content of nucleus pulposus in rats (n = 3). (H) Cell viability of primary rat NP cells subjected to different time length compression (n = 3). (I, J) The protein expression levels of Aggracan, Collagen II, MMP3, and MMP13 in primary rat NP cells after being compressed for different time periods, as determined by Western blotting (n = 3). (K) Changes in morphology and cytoskeleton of primary NP cells in rats after compression. Data are represented as mean ± SD. p value was calculated with t‐test or ANOVA. *p < 0.05, **p < 0.01, ***p < 0.001. Figure S2: (A, B) IF staining of Vim in coccygeal IVDs in rats (n = 3). Scale bar: 200 μm. (C, D) The mRNA expression levels of Vimentin in primary rat NP cells in each group was determined by qRT‐PCR (n = 3). (E, F) The protein expression levels of Vimentin in primary rat NP cells in each group was determined by Western blotting. (G, H) The proteoglycan content in NP cells of each group was detected by Alcian blue staining (n = 3). Scale bar: 50 μm. (I, J) The protein expression levels of Aggracan, Collagen II, MMP3, and MMP13 in primary rat NP cells determined by Western blotting (n = 3). (K, L) The proteoglycan content in NP cells of each group was detected by Alcian blue staining (n = 3). Scale bar: 50 μm. Data are represented as mean ± SD. p value was calculated with t‐test or ANOVA. *p < 0.05, **p < 0.01, ***p < 0.001. Figu [file CPR-9999-e70256-s001.docx]

**Table S1.** The primary antibodies for IHC, IF, and western blot.

| Antibody | Dilution ratio  （for IHC） | Dilution ratio  （for IF of NP） | Dilution ratio  （for IF of cells） | Dilution ratio  （for western blot） | Manufacturer |
| --- | --- | --- | --- | --- | --- |
| Anti-Aggrecan | - | - | - | 1:1000 | Affinity（DF7561） |
| Anti-Collagen II | 1:500 | 1:200 | 1:200 | 1:1000 | Proteintech（28459-1） |
| Anti-MMP 3 | 1:100 | 1:200 | 1:200 | 1:1000 | Huabio（JM46-22） |
| Anti-MMP 13 | - | - | - | 1:1000 | Proteintech（18165-1） |
| Anti-NLRP3 | 1:200 | 1:200 | 1:250 | 1:2000 | Proteintech（68102-1） |
| Anti-GSDMD | 1:100 | 1:100 | 1:100 | 1:1000 | Abclonal（A24476） |
| Anti-Caspase-1 | - | - | - | 1:1000 | Abmart（P79884R2） |
| Anti-IL-1β | 1:100 | 1:100 | 1:100 | 1:1000 | Abmart（P50520-1） |
| Anti-Vimentin | 1:100 | 1:300 | 1:500 | 1:1000 | Abmart（T55134） |
| Anti-LC3B | 1:100 | 1:200 | 1:200 | 1:1000 | Abmart（T55992） |
| Anti-P62 | 1:100 | 1:200 | - | 1:5000 | Abmart（T55546） |
| Anti-PINK1 | 1:1000 | 1:2000 | - | 1:2000 | Proteintech（23274-1） |
| Anti-Tom20 | - | - | 1:100 | - | Abmart（T55527） |
| Anti-Parkin | 1:200 | 1:200 | 1:200 | 1:2000 | Proteintech（14060-1） |
| Anti-Itgb1 | 1:1000 | - | 1:200 | 1:5000 | Proteintech（12594-1） |
| Anti-MNAT1 | - | - | 1:400 | 1:5000 | Proteintech（11719-1） |
| Anti-Ubiquitin | - | - | - | 1:3000 | Proteintech（10201-2） |
| Anti-β-actin | - | - | - | 1:1000 | ZSGB（TA-09） |


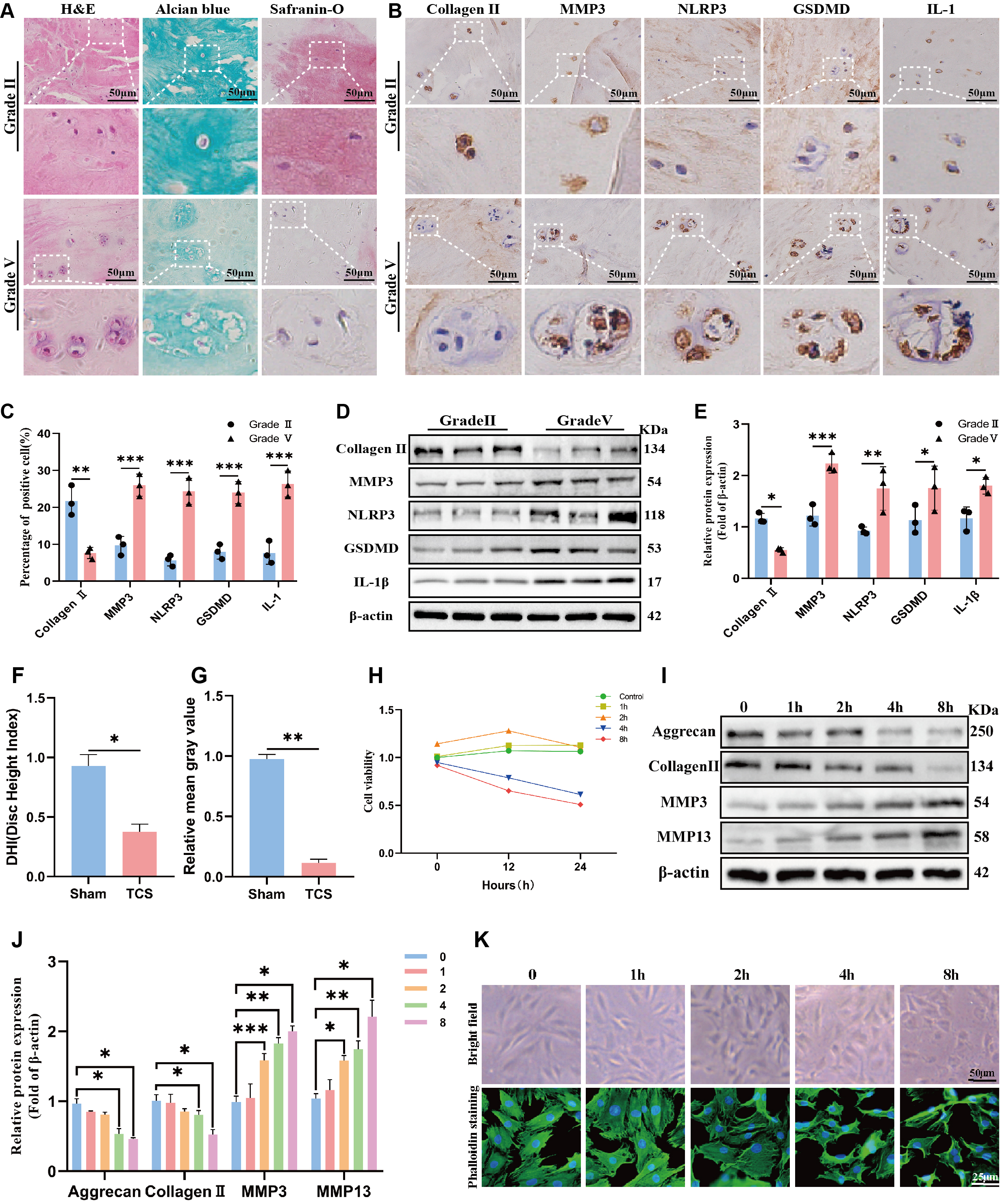
**Figure S1**. (A) H&E, Alcian blue, and Safranin-O staining of human NP samples. Scale bar: 50 μm. (B, C) Immunohistochemical staining of Collagen Ⅱ, MMP3, NLRP3, GSDMD, and IL-1β in different degenerative human NP tissues (n=3). Scale bar: 50 μm. (D, E) The protein expressions of ECM (Collagen Ⅱ, MMP3) and pyroptosis indicators (NLRP3, GSDMD, and IL-1β) in different degenerative NP tissues, as determined by western blotting (n=3). (F) Quantitative analysis of intervertebral disc height in rats (n=3). (G) Quantitative analysis of water content of nucleus pulposus in rats (n=3). (H) Cell viability of primary rat NP cells subjected to different time length compression (n=3). (I, J) The protein expression levels of Aggracan, Collagen II, MMP3, and MMP13 in primary rat NP cells after being compressed for different time periods, as determined by Western blotting (n=3). (K) Changes in morphology and cytoskeleton of primary NP cells in rats after compression. Data are represented as mean ± SD. *P* value was calculated with t-test or ANOVA. **p* < 0.05, ***p* < 0.01, ****p* <0.001.


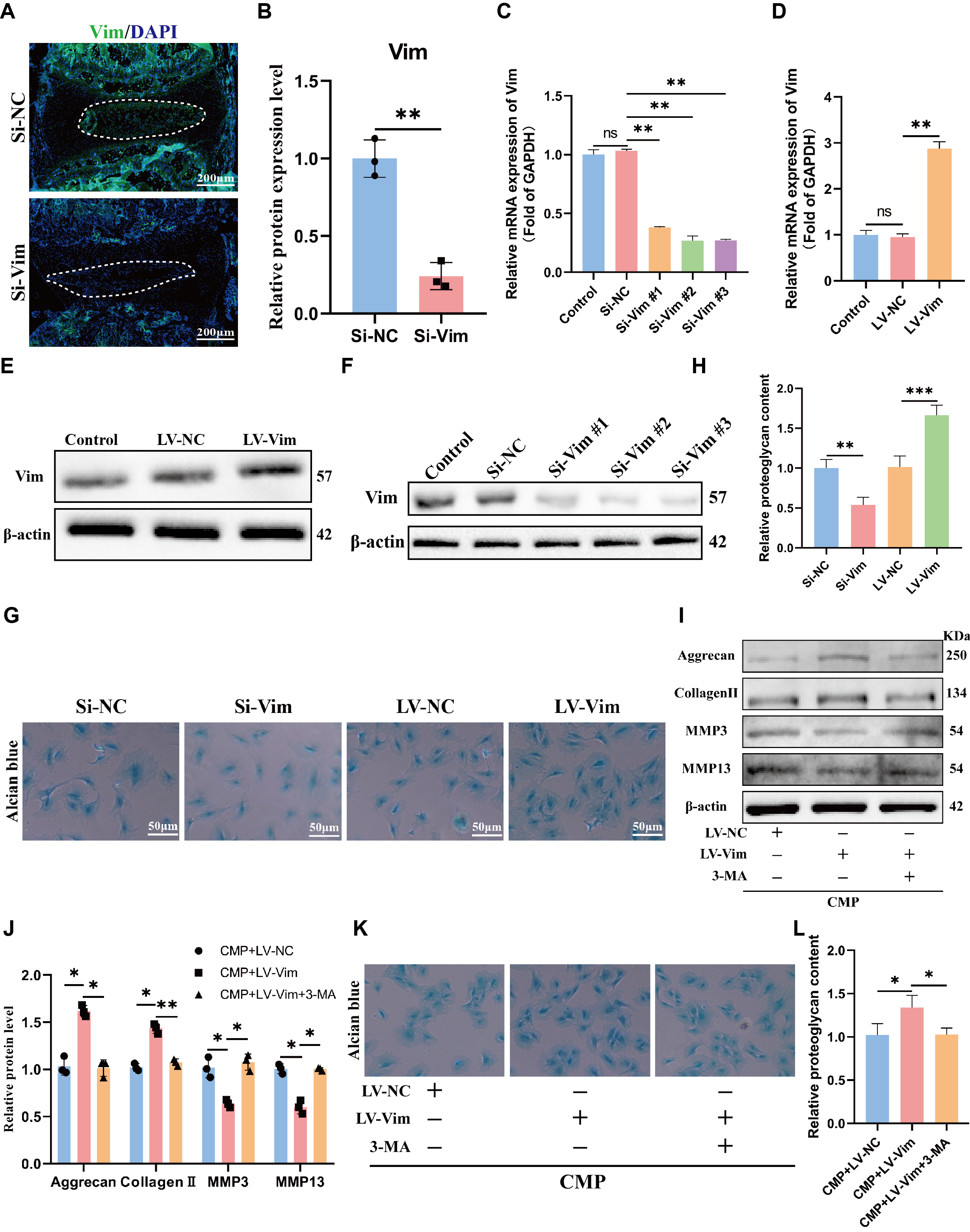


**Figure S2**. (A, B) IF staining of Vim in coccygeal IVDs in rats (n=3). Scale bar: 200 μm. (C, D) The mRNA expression levels of Vimentin in primary rat NP cells in each group was determined by qRT-PCR (n=3). (E, F) The protein expression levels of Vimentin in primary rat NP cells in each group was determined by Western blotting. (G, H) The proteoglycan content in NP cells of each group was detected by Alcian blue staining (n=3). Scale bar: 50 μm. (I, J) The protein expression levels of Aggracan, Collagen II, MMP3, and MMP13 in primary rat NP cells determined by Western blotting (n=3). (K, L) The proteoglycan content in NP cells of each group was detected by Alcian blue staining (n=3). Scale bar: 50 μm. Data are represented as mean ± SD. *P* value was calculated with t-test or ANOVA. **p* < 0.05, ***p* < 0.01, ****p* <0.001.


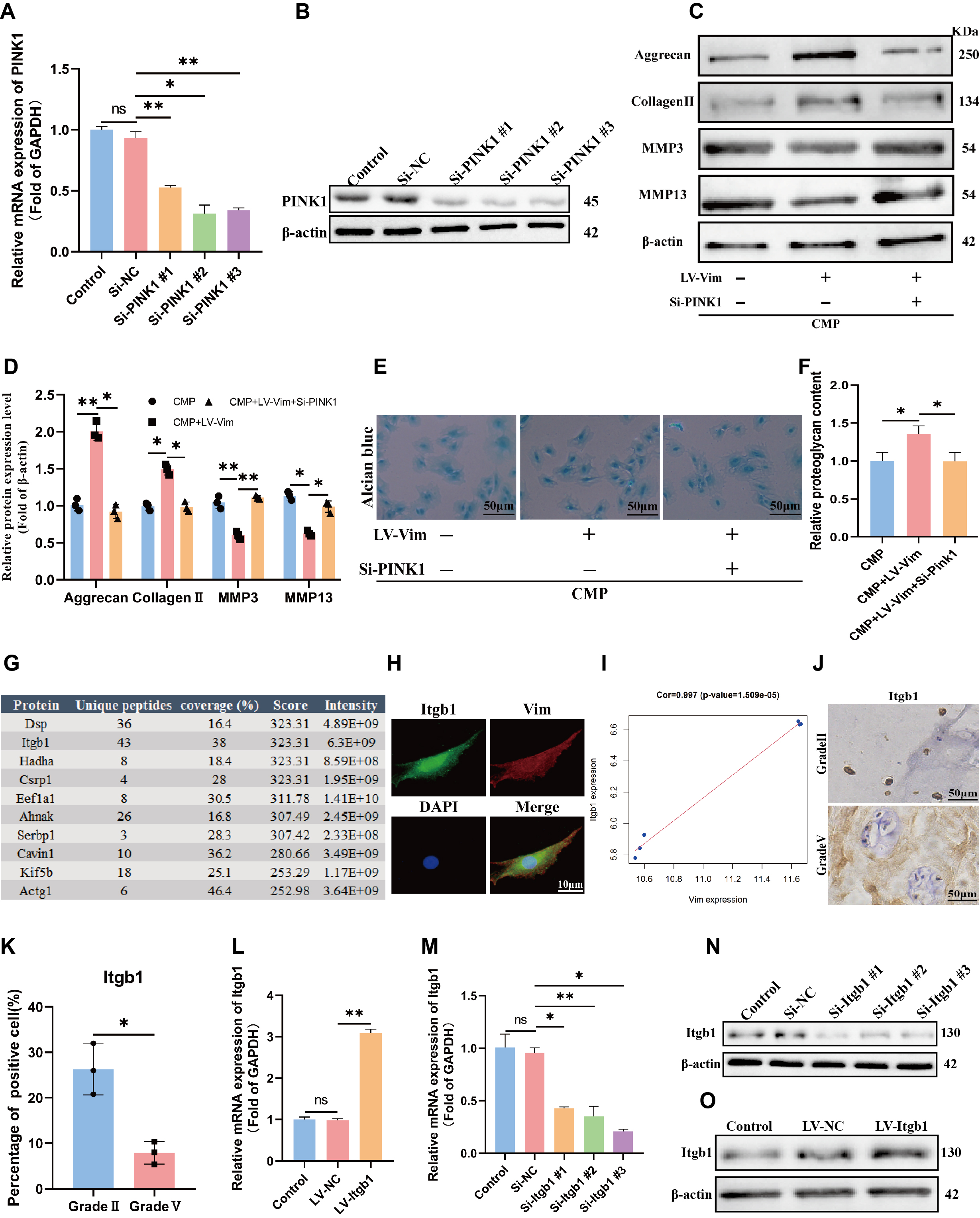
**Figure S3**. (A) The mRNA expression levels of PINK1 in primary rat NP cells in each group was determined by qRT-PCR (n=3). (B) The protein expression levels of PINK1 in primary rat NP cells in each group was determined by Western blotting. (C, D) The protein expression levels of Aggracan, Collagen II, MMP3, and MMP13 in primary rat NP cells determined by Western blotting (n=3). (E, F) The proteoglycan content in NP cells of each group was detected by Alcian blue staining (n=3). Scale bar: 50 μm. (G) IP-MS analysis revealed the presence of Itgb1. (H) Immunofluorescence colocalization of Itgb1 and Vimentin. Scale bar: 10 μm. (I) The correlation between Itgb1 and Vimentin expression levels in the GSE266883 dataset. (J, K) Immunohistochemical staining of Itgb1 in different degenerative human NP tissues (n=3). Scale bar: 50 μm. (L, M) The mRNA expression levels of Itgb1 in primary rat NP cells in each group was determined by qRT-PCR (n=3). (N, O) The protein expression levels of Itgb1 in primary rat NP cells in each group was determined by Western blotting. Data are represented as mean ± SD. *P* value was calculated with t-test or ANOVA. **p* < 0.05, ***p* < 0.01, ****p* <0.001.


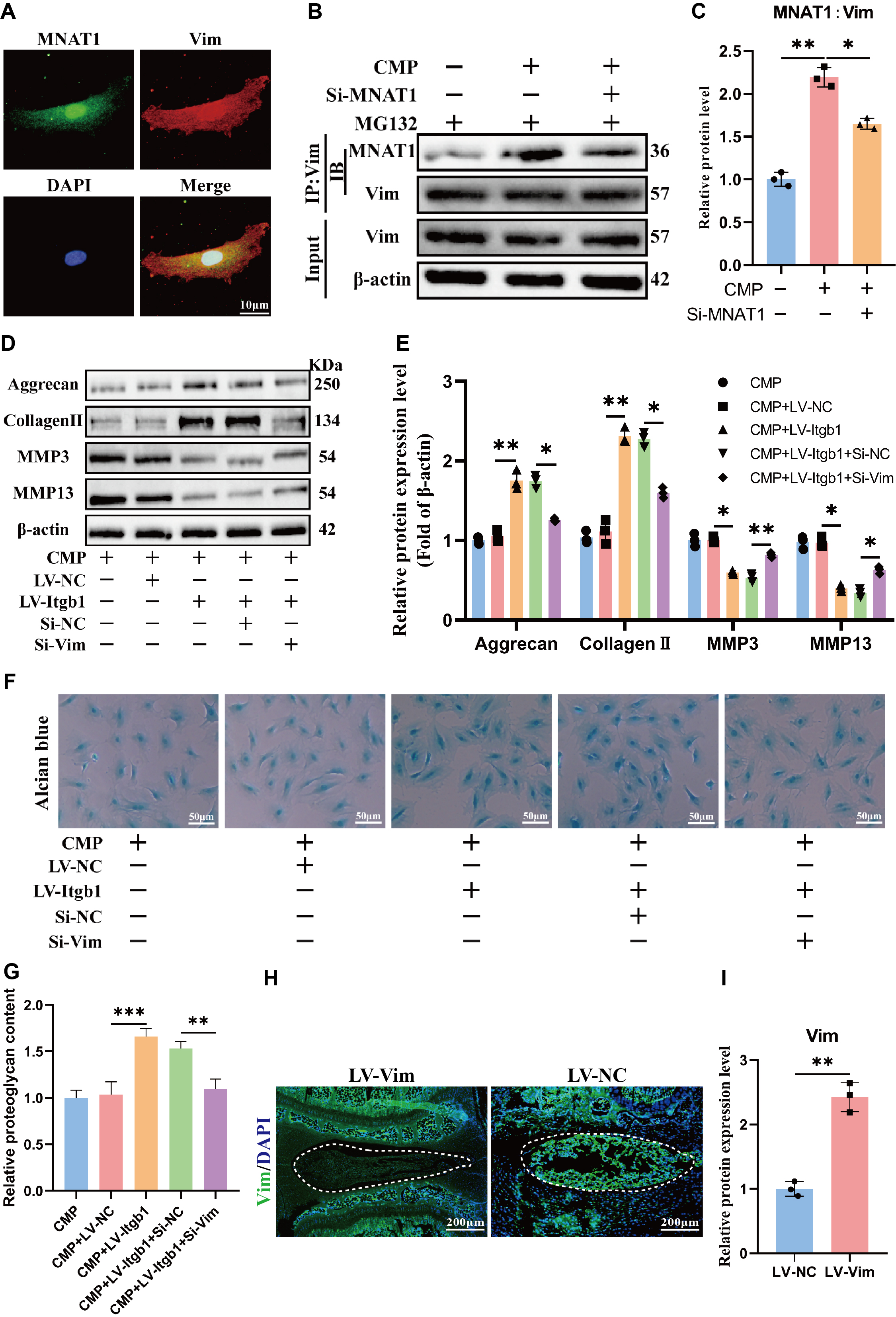
**Figure S4**. (A) Immunofluorescence colocalization of MNAT1 and Vimentin. Scale bar: 10 μm. (B, C) The binding levels of MNAT1 and Vimentin in compressed primary rat NP cells through IP analysis. IP with anti-Vimentin antibody (n=3). (D, E) The protein expression levels of Aggracan, Collagen II, MMP3, and MMP13 in primary rat NP cells determined by Western blotting (n=3). (F, G) The proteoglycan content in NP cells of each group was detected by Alcian blue staining (n=3). Scale bar: 50 μm. (H, I) IF staining of Vim in coccygeal IVDs in rats (n=3). Scale bar: 200 μm. Data are represented as mean ± SD. *P* value was calculated with t-test or ANOVA. **p* < 0.05, ***p* < 0.01, ****p* <0.001.
